# *GmWRKY21*, a Soybean WRKY Transcription Factor Gene, Enhances the Tolerance to Aluminum Stress in *Arabidopsis thaliana*

**DOI:** 10.3389/fpls.2022.833326

**Published:** 2022-07-25

**Authors:** Zhenzhen Han, Jinyu Wang, Xinxin Wang, Xijia Zhang, Yanbo Cheng, Zhandong Cai, Hai Nian, Qibin Ma

**Affiliations:** ^1^The State Key Laboratory for Conservation and Utilization of Subtropical Agro-Bioresources, South China Agricultural University, Guangzhou, China; ^2^The Key Laboratory of Plant Molecular Breeding of Guangdong Province, College of Agriculture, South China Agricultural University, Guangzhou, China; ^3^The Guangdong Subcenter of the National Center for Soybean Improvement, College of Agriculture, South China Agricultural University, Guangzhou, China; ^4^The Guangdong Provincial Laboratory of Lingnan Modern Agricultural Science and Technology, South China Agricultural University, Guangzhou, China; ^5^Zengcheng Teaching and Research Bases, South China Agricultural University, Guangzhou, China

**Keywords:** transcription factor, *GmWRKY21*, soybean, Al stress, *Arabidopsis thaliana*

## Abstract

The WRKY transcription factors (TFs) are one of the largest families of TFs in plants and play multiple roles in plant growth and development and stress response. In this study, *GmWRKY21* encoding a WRKY transcription factor was functionally characterized in Arabidopsis and soybean. The GmWRKY21 protein containing a highly conserved WRKY domain and a C_2_H_2_ zinc-finger structure is located in the nucleus and has the characteristics of transcriptional activation ability. The *GmWRKY21* gene presented a constitutive expression pattern rich in the roots, leaves, and flowers of soybean with over 6-fold of relative expression levels and could be substantially induced by aluminum stress. As compared to the control, overexpression of *GmWRKY21* in Arabidopsis increased the root growth of seedlings in transgenic lines under the AlCl_3_ concentrations of 25, 50, and 100 μM with higher proline and lower MDA accumulation. The results of quantitative real-time polymerase chain reaction (qRT-PCR) showed that the marker genes relative to aluminum stress including *ALMT, ALS3, MATE*, and *STOP1* were induced in *GmWRKY21* transgenic plants under AlCl_3_ treatment. The stress-related genes, such as *KIN1, COR15A, COR15B, COR47, GLOS3*, and *RD29A*, were also upregulated in *GmWRKY21* transgenic Arabidopsis under aluminum stress. Similarly, stress-related genes, such as *GmCOR47, GmDREB2A, GmMYB84, GmKIN1, GmGST1*, and *GmLEA*, were upregulated in hair roots of *GmWRKY21* transgenic plants. In summary, these results suggested that the GmWRKY21 transcription factor may promote the tolerance to aluminum stress mediated by the pathways regulating the expression of the acidic aluminum stress-responsive genes and abiotic stress-responsive genes.

## Introduction

Aluminum (Al) mainly existing in the forms of harmless oxides and aluminosilicates is the third most abundant metal in the earth's crust next to oxygen and silicon (Kochian et al., 2004). Acidic soils are widespread with ~30% of the world's land area and about 50% of the potential of the world's potentially arable lands (Silambarasan et al., 2019). In acidic soil (pH <5), Al^3+^ ions are activated and dissolved into the soil solution from clay minerals, which can quickly inhibit root growth resulting in reduced crop yield (Kochian et al., 2015). Therefore, Al toxicity is the main factor restricting plant growth in acidic soil (Bojorquez-Quintal et al., 2017). The solubility of aluminum increases with the decrease in pH value (Brautigan et al., 2012). In an acidic solution, Al^3+^ exists as the octahedral hexahydrate ${\text{Al (H}}_{2}{\text{O)}}_{6}^{3+}$ commonly known as Al^3+^ (Kochian et al., 2015), while the Al toxicity in acidic soil is mainly caused by Al^3+^(Singh et al., 2017). The most apparent feature of Al toxicity is to inhibit the root tip cell elongation and cell division. It was generally believed that the root tip is the central part of Al toxicity (Kochian, 1995). The transition zone (DTZ) of the root tip 1–2 mm between the meristematic zone and the elongation zone is the most sensitive area for aluminum poisoning (Kollmeier et al., 2000). Al^3+^ is electrostatically bound to the pectin of the negatively charged root tip cell wall (Kochian, 1995), which reduces the rate of cell division and extension and affects root elongation.

Previous studies showed that the higher concentration of Al^3+^ will cause severe toxicity to plants because it will inhibit cell elongation and cell division in the roots, which will lead to enlarged root tips, reduced root hairs, or no root hair development, thus hindering the absorption of nutrients and water (Kochian et al., 2015). Al^3+^ toxicity increases the formation of reactive oxygen species (ROS) in plants by leading to lipid membrane peroxidation, ion leakage, protein oxidation, callose accumulation, and cell death (Ezaki et al., 2000; Yamamoto et al., 2002; Boscolo et al., 2003).

In adaptation to Al stress, plants have evolved two resistance mechanisms: external and internal tolerance mechanisms (Liu et al., 2014; Kochian et al., 2015; Wang N. et al., 2017). Several main components exist in the external mechanism including the cell wall of Al^3+^ fixation, induced pH increase, organic acid (OA) anions chelate Al^3+^ ions, and Al^3+^ transmembrane outflow, while some main components are involved in the internal tolerance mechanism including plants chelate aluminum ions through organic acids and proteins in cells and transporting aluminum ions from sensitive cytoplasmic areas to non-sensitive areas, such as vacuoles (Feng, 2000; Ryan et al., 2001; Kochian et al., 2015; Silambarasan et al., 2019). Aluminum-activated malate transporter (ALMT) and multidrug and toxic compound extrusion (MATE) identified in plants are two transporter families that confer Al tolerance through the secretion of the organic acids. *TaALMT1* was the first identified malate efflux gene in wheat (Sasaki et al., 2004). Another organic acid efflux transporter family MATEs as a plasma membrane efflux transporter are responsible for the Al-activated citrate release (Liu et al., 2009, 2012).

Transcription factors are proteins that regulate gene transcription to adapt to the environmental stimulus (Finatto et al., 2018). WRKY proteins have one or two conserved WRKY domains of about 60 amino acids with an invariable sequence motif WRKYGQK at the N-terminus and a zinc-finger domain-like (CX_4−5_CX_22−23_HXH or CX_7_CX_23_HXC) (Eulgem et al., 2000; Rushton et al., 2010). The WRKY TFs can be classified into three main groups (I–III) based on the number of WRKY domains and the type of their zinc-finger motifs. The WRKY TFs in group I have two WRKY domains and a C_2_H_2_-type zinc-finger structure. The TFs in group II have a single WRKY domain and contain the zinc-finger motif C_2_H_2_, which is divided into five subgroups (IIa–e). The TFs in group III hold a WRKY domain and contain a C_2_-HC motif (Eulgem et al., 2000; Zhang and Wang, 2005; Song et al., 2018; Xie et al., 2018).

WRKY is a superfamily of transcription factors in plants that play an essential role in many life processes, especially in biotic and abiotic stresses (Eulgem et al., 2000; Zhang and Wang, 2005; Rushton et al., 2010; Chen et al., 2012; Li et al., 2013). *SPF1* was the first WRKY gene isolated from sweet potato in 1994 (Ishiguro and Nakamura, 1994), and then, the *ABF1* and *ABF2* genes were discovered in wild oats (Rushton et al., 1995). The genes of *WRKY1, WRKY2*, and *WRKY3* from parsley were confirmed for the first time that WRKY protein could regulate the response of plants to pathogens (Rushton et al., 1996).

In Arabidopsis, overexpression of *AtWRKY25* and *AtWRKY33* increased salt tolerance in transgenic plants (Jiang and Deyholos, 2009). Overexpression of *AtWRKY57, ABO3, AtWRKY70, AtWRKY46*, and *AtWRKY54* in Arabidopsis conferred the tolerance of transgenic plants to drought stress (Ren et al., 2010; Jiang et al., 2012; Chen et al., 2017). Overexpression of *GhWRKY34* enhanced the tolerance of transgenic Arabidopsis to salt stress by selectively taking up Na^+^ and K^+^ (Zhou et al., 2015). *GhWRKY1-like* encodes a WRKY TF-mediated drought tolerance in Arabidopsis *via* modulating ABA biosynthesis (Hu et al., 2021). Besides these, *AtWRKY3* and *AtWRKY4* increased salt stress tolerance in transgenic Arabidopsis (Li C. et al., 2021). In rice, overexpression of *OsWRKY11* controlled by the *HSP101* promoter resulted in the enhancement of drought resistance, which was characterized by slower leaf wilting and a higher survival rate (Wu et al., 2009). *OsWRKY30* in transgenic rice enhanced the tolerance to drought stress (Shen et al., 2012). Overexpression of *OsWRKY45* and *OsWRKY72* can change the drought resistance of transgenic Arabidopsis, which may be related to the induction of genes related to abscisic acid stress (Qiu and Yu, 2009; Song et al., 2010). Recent studies have shown that OsWRKY108 was an integrative regulator of P homeostasis and leaf inclination (Wang et al., 2021). The inactivated *OsWRKY5* enhanced drought tolerance through abscisic acid signaling pathways (Lim et al., 2021). At 4°C, overexpression of *OsWRKY76* improved the tolerance to low-temperature stress (Babitha et al., 2013). OsWRKY50 enhanced salt stress tolerance *via* an ABA-independent pathway (Huang et al., 2021). In wheat, *TaWRKY2* overexpression improved salt and drought tolerance by reducing the expression of *STZ* and *RD29B* (Niu et al., 2012). *TaWRKY146* enhanced drought tolerance by inducing stomatal closure in Arabidopsis (Ma et al., 2017). *TaWRKY1* and *TaWRKY33* made transgenic Arabidopsis resistant to drought and high-temperature stress (He et al., 2016). The heterologous transgenic plants of the *TaWRKY19* gene had salt and drought tolerance and showed tolerance to low temperatures (Niu et al., 2012). *TaWRKY74* participated in copper tolerance through regulation of *TaGST1* expression and GSH content (Li G. Z. et al., 2021). Ectopic expression of *TaWRKY75-A* improved drought and salt resistance in transgenic Arabidopsis (Ye et al., 2021). Among the WRKY genes in soybean, overexpression of *GmWRKY21* enhanced the tolerance to cold stress in Arabidopsis (Zhou et al., 2008). *GmWRKY54* and *GmWRKY49* enhanced the tolerance to salt and drought stress in transgenic Arabidopsis (Zhou et al., 2008; Xu et al., 2018), whereas *GmWRKY16* conferred salt and drought tolerance (Ma et al., 2019). Overexpression of *GmWRKY12* increased the tolerance of transgenic soybean seedlings to drought and salt stress by proline accumulation and malondialdehyde decrease. *GmWRKY46* overexpression showed that it was involved in hairy root development and subsequently affected plant growth and Pi uptake (Li C. et al., 2021). It was found that the research progresses on the functions of WRKY family genes in soybean mostly focused on the abiotic stress response. However, there are few reports on the functions of WRKY family genes in acidic aluminum stress in soybean. In this study, *GmWRKY21*, an abiotic stress-responsive gene induced by Al stress, was investigated for its functional characterization of Al stress tolerance both in Arabidopsis and in soybean. The systems of Arabidopsis genetic transformation and soybean hairy root transformation were used to study the resistance and molecular mechanism of *GmWRKY21* responding to acidic aluminum stress.

## Materials and Methods

### Plant Materials and Stress Treatments

A soybean cultivar, “Huaxia 3” (HX3), bred by the Guangdong Subcenter of the National Center for Soybean Improvement in South China Agricultural University, was used to investigate the tissue expression pattern of *GmWRKY21* and its responses to Al stress. Soybean seeds of HX3 were germinated in vermiculite with room temperature set at 28/26°C and the light time set as 14-h light/10-h dark under a light intensity of 100 μmol/(m^2^·s) (Zeng et al., 2012). For the Al dose–response experiment, seedlings were subjected to a modified 0.5 mM CaCl_2_ solution containing 0, 25, 50, 75, or 100 μM of AlCl_3_ for 24 h. During the time-course experiment, seedlings were subjected to a modified 0.5 mM CaCl_2_ solution with 50 μM AlCl_3_ for 0, 6, 9, 12, 24, 36, or 48 h.

### Plasmid Construction and Transformation of *GmWRKY21* in *Arabidopsis*

The cDNA sequences of *GmWRKY21* gene were isolated using specific primers (Supplementary Table 1) and inserted into the multicloning sites of the pLB vector to form the *GmWRKY21*-pLB construct. Then, the full-length coding sequence of *GmWRKY21* amplified from the *GmWRKY21*-pLB vector was inserted into the *Sac*I and *Xba*I sites of a pTF101.1 vector. The non-conserved region sequence 200 bp of *GmWRKY21* amplified by PCR using the specific primers was inserted into the *Avr*II and *Asc*I sites, and then, the 200-bp sequence was reversely inserted into the *Sac*I and *Spe*I sites of a pMU103 vector using the ClonExpressR II One Step Cloning Kit (C112, Vazyme, Nanjing, China). The *GmWRKY21*-pTF101 and *GmWRKY21*-pMU103 plasmids were then transformed into *Agrobacterium* strains, GV3101 and K599 competent cells by heat shock, respectively. The subsequent transformations were carried out by the methods of the *Agrobacterium*-mediated floral dip (Clough and Bent, 2010) and *Agrobacterium rhizogenes*-based transformation (Matthews and Youssef, 2016).

### The Isolation and Sequence Analysis of the *GmWRKY21* Gene

The sequence information of the *GmWRKY21* gene was obtained from the database of the National Center for Biotechnology Information (NCBI) with the accession number NP_001237327.2 (Wang et al., 2019). The full-length sequence of *GmWRKY21* was amplified by RT-PCR using specific primers (Supplementary Table 1) and Super-Fidelity DNA polymerase (Phanta Max, Vazyme Biotech Co., Ltd., Nanjing, China). The seedlings of soybean cultivar HX3 under the treatment of 50 μM AlCl_3_ (1 mM CaCl_2_, pH 4.5) were used to extract total RNA by TRIzol reagent (Invitrogen). The methods of generated cDNA, RT-PCR, and agarose gel electrophoresis were described in detail previously (Lu et al., 2015). The purified PCR product was then inserted into the multiple cloning sites of the pLB vector (Tiangen Rapid DNA Ligation Kit, Beijing, China). The positive clones in *Escherichia coli* were used to obtain the full cDNA sequence of *GmWRKY21* identified by PCR, enzyme digestion, and sequencing (Sangon Biotech Co., Ltd., Shanghai, China) (Lu et al., 2015; Ma et al., 2018).

The nucleotide sequence of *GmWRKY21* and the amino acid sequence of GmWRKY21 protein were used to search its homologous genes and proteins using the database of NCBI. The alignment of nucleotide sequences was performed with the software of DNAMAN. The structure prediction of the GmWRKY21 protein was carried out using the NCBI blast results. The phylogenetic analysis was enforced by using the software of MEGA X (Kumar et al., 2016). The alignment was adjusted manually, while the unrooted phylogenetic trees were constructed by the neighbor-joining method (Sievers et al., 2011).

### Intracellular Localization of *GmWRKY21* Protein

Localization of the ‘protein was carried out using the method described previously (Lu et al., 2015). The complete coding sequence of *GmWRKY21* without a stop codon amplified by PCR using the specific primers was inserted into the *Nco*I and *Spe*I sites to generate a fusion construct of the GmWRKY21-pCAMBIA1302 vector under the control of CaMV 35S promoter. The plasmids of pCAMBIA1302 and GmWRKY21-pCAMBIA1302 were transformed into *Agrobacterium tumefaciens* strain GV3101 by the heat shock method. The young leaves of 4-week-old tobacco plants were contaminated by the recombinant Agrobacterium tumefaciens using the method described previously in detail (Kokkirala et al., 2010). The agro-infiltration leaves of tobacco plants cultured for 48h to 72 h were photographed with a confocal laser scanning microscope (Leica, Germany).

### Transactivation Assay of *GmWRKY21* Protein

The ORF sequence of *GmWRKY21* amplified by PCR using the specific primers was inserted into the *Eco*RI and *Bam*HI sites to create a fusion construct of GmWRKY21-pGBKT7 (Supplementary Table 1). The GmWRKY21-pGBKT7 and pGBKT7 plasmids were transformed into the cells of yeast strain Y2H. Transcriptional activation of GmWRKY21 protein was analyzed according to the methods described previously (Yang et al., 2010).

### Expression Analysis by qRT-PCR

Total RNA was extracted from the seedlings of soybean or Arabidopsis using the Plant Total RNA Kit (GeneMark, Taiwan, China). Reversing transcription for the first-strand cDNA synthesis was performed with 2 μg of total RNA using a PrimeScriptTM RT reagent Kit (Takara, Beijing, China). The qRT-PCR analysis was performed on a CFX96TM Real-Time System (Bio-Rad, Hercules, CA, USA) with SYBR Premix Ex Taq II (Takara, Beijing, China). The inner reference gene *Actin* 3 was used to normalize the data. The quantitative variations of gene expression between the examined replicates were evaluated by the 2^−ΔΔCt^ method described previously (Willems et al., 2008; Lu et al., 2015). Three independent biological repeats were performed to ensure accurate statistical analysis. The specific primers are listed in Supplementary Table 1.

### Acidic Aluminum Treatment in Transgenic Arabidopsis

Two homozygous lines of T_3_ generation of *GmWRKY21* transgenic plants were used for Al sensitivity assay by measuring relative root elongation according to the method described previously (Yokosho et al., 2016). After seed germination in the MS medium, 5-day-old seedlings of wild-type and *GmWRKY21* transgenic plants were transferred to a solid agar medium supplied with 1 mM CaCl_2_ and 1% sucrose containing different concentrations of AlCl_3_ (0, 25, 50, and 100 μM; pH 4.5). To prepare 100 mM of AlCl_3_ stock solution, 2.414 g of AlCl_3_·6H_2_O was dissolved in 100 ml of distilled water and sterile-filtered. The root lengths were measured before and after 3 days of different treatments. The relative root elongation (RRE) was computed as the formula, that is, RRE = (root elongation with different treatments/root elongation without treatments) ×100 (Cai et al., 2020).

### Hair Root Transformation in Soybean

The plasmids of the empty vector (EV), *GmWRKY21*-pTF101 (OE), and *GmWRKY21*-pMU103 (RNAi) constructs were transformed into the competent cells of *Agrobacterium* strains K599 by heat shock. Hairy root transformation was performed according to previous reports (Kereszt et al., 2007; Ren et al., 2019). Hypocotyls of 5-day-old soybean seedlings were injected with *Agrobacterium rhizogenes* K599 and kept at high humidity (>90%) with plastic lids until hairy roots at the injection sites were developed to 5–10 cm long. Then, the main roots of the original seedlings were cut, and the hairy roots were immersed in water for ~5 days before the plants with hairy roots were transferred into the solutions of 0 and 50 μM AlCl_3_ (0.5 mM CaCl_2_, pH 4.3) for 24 h.

### Data Analysis

All data were presented as the mean of three biological replicates ± SEM. Student's *t*-test at *P* = 0.01 or *P* = 0.05 was used to identify the difference between observation values (Lu et al., 2015).

## Results

### Cloning and Bioinformatics Analysis of *GmWRKY21*

Based on recent reports of QTL mapping for the aluminum tolerance of soybean (Wang et al., 2019), an aluminum stress-induced gene encoding a transcription factor of WRKY protein was obtained from the database under the gene locus GLYMA_04G218700 and the protein accession number of NP_001237327.2. The WRKY gene designed as *GmWRKY21* is located on chromosome 4 of soybean. The *GmWRKY21* open reading frame (ORF) was isolated from the soybean variety Huaxia 3 based on putative sequence information available from the Phytozome database. The sequence analysis showed that the *GmWRKY21* gene contained three exons and two introns, which encoded a 196-amino-acid protein with an estimated molecular mass of 22.521 KDa and an isoelectric point of 6.143 (data not shown).

**Figure 1:**
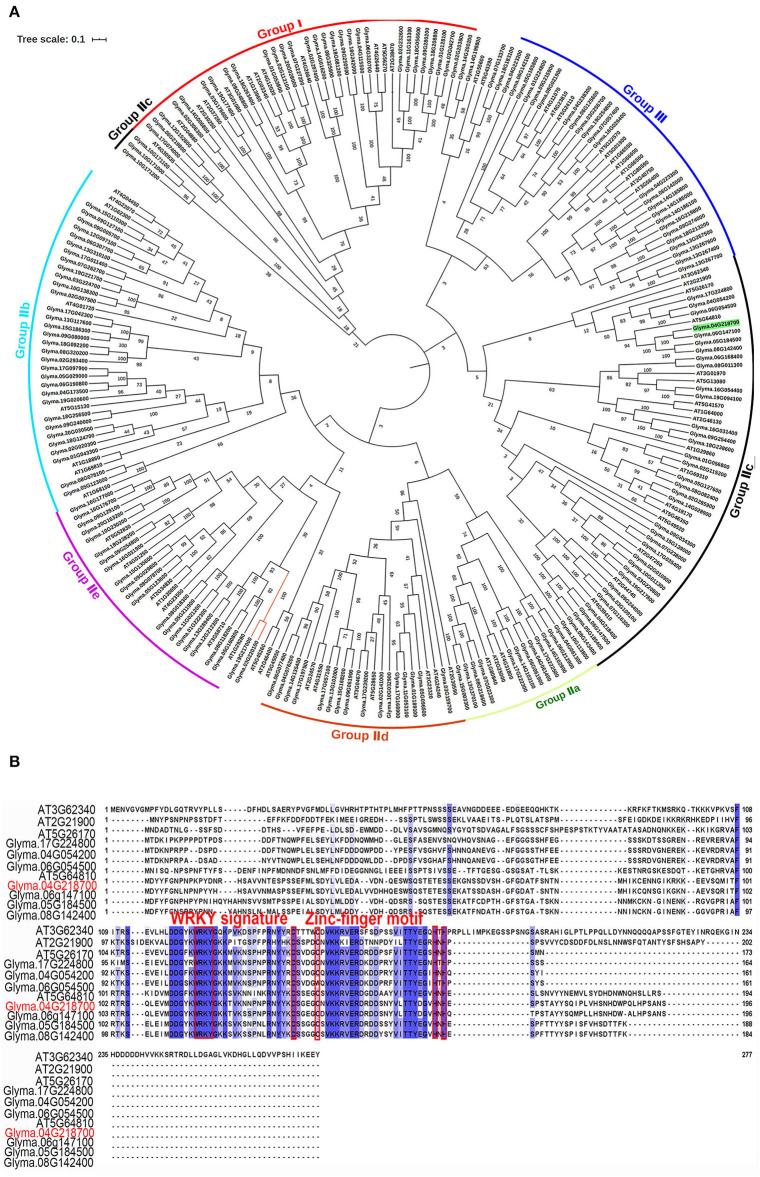
Bioinformatics analysis of the *GmWRKY21* gene. **(A)** Phylogenetic tree was constructed to analyze GmWRKY21 and WRKY proteins in soybean and Arabidopsis by the neighbor-joining method using the software of MEGA X. We selected 177 and 71 WRKT family genes in soybean and *Arabidopsis*, respectively (Supplementary Table 1). The amino acid sequences and the accession numbers of WRKY proteins from soybean and Arabidopsis were obtained from the databases of NCBI (https://www.ncbi.nlm.nih.gov/). **(B)** Multiple sequence alignment of genes belonged to the same branch of GmWRKY21 from the phylogenetic tree in **(A)**. Red box highlights the WRKY domains and the zinc-finger motifs.

The protein BLAST analysis using the complete amino acid sequences indicated that the GmWRKY21 protein had a WRKY DNA-binding domain at the location of the peptide chain between 111 aa and 168 aa. The relationship analysis between GmWRKY21 and other WRKY proteins in soybean and Arabidopsis by the phylogenetic tree showed that *Glyma.06g147100* was 91.3% sequence similar to its homology of the *GmWRKY21* gene in soybean (Figure 1A), while *AT5G64810* with the highest homology in Arabidopsis was 40.8% sequence similar to that of the *GmWRKY21* gene. The results of multiple sequence alignment indicated there were three groups for all the WRKY proteins with the largest subgroup of group II. The GmWRKY21 protein was a member of group IIc containing a typical WRKY domain and a C_2_H_2_ zinc-finger motif (Eulgem et al., 2000; Figure 1B). In group IIc, some genes had higher sequence similarity to that of *GmWRKY21*. Among them, *GmWRKY6* held the tolerance to salt and drought stress (Zhou et al., 2008). The results suggested that GmWRKY21 protein was a WRKY TF in soybean which may play certain roles in responding to abiotic stress.

### Characteristics of Localization and Transcriptional Activation Ability of *GmWRKY21* Protein

**Figure 2:**
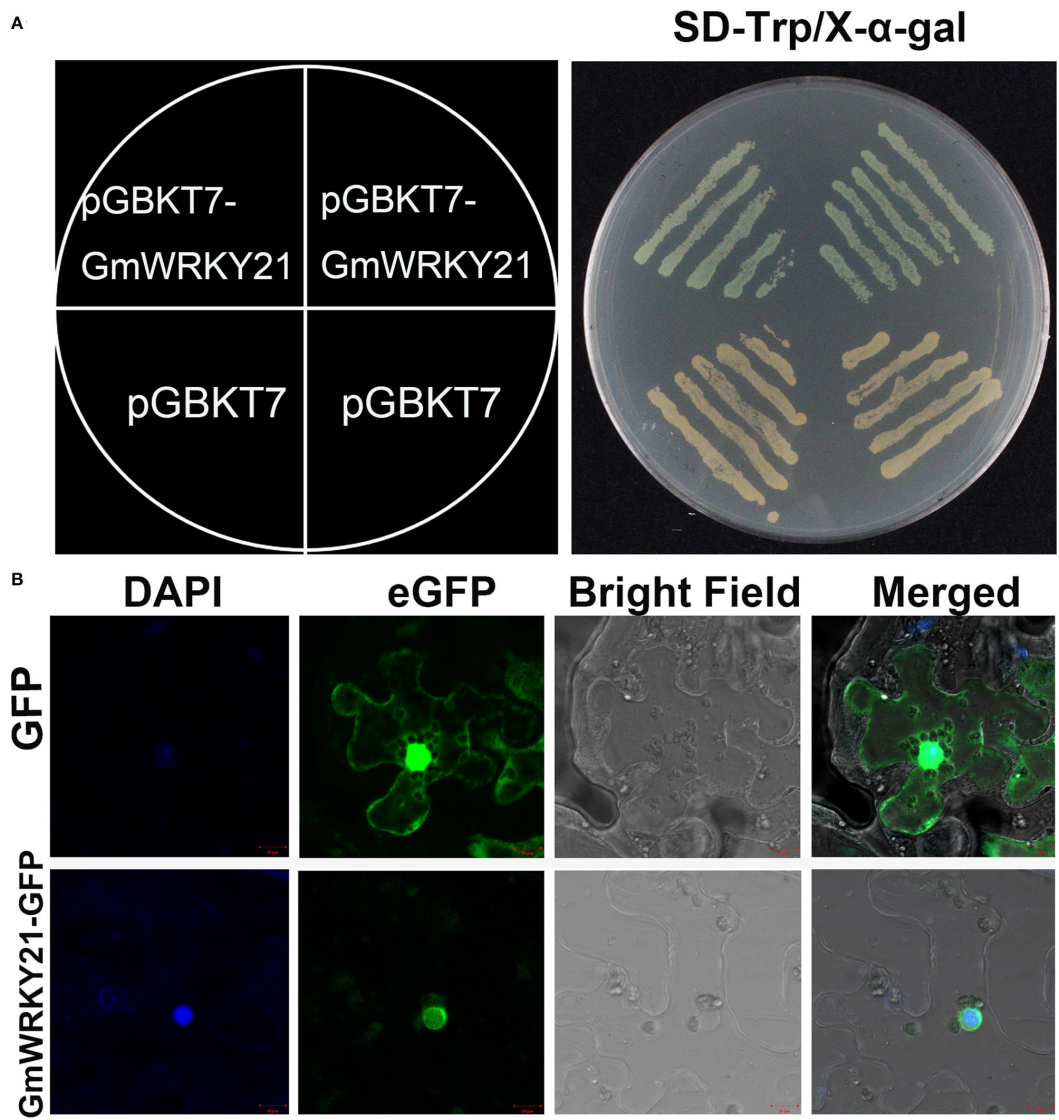
Transcriptional activation and subcellular localization of GmWRKY21 protein. **(A)** Transcriptional activation analysis of GmWRKY21 protein. **(B)** Subcellular localization of GmWRKY21 protein in leaf epidermal cells of tobacco. The ORF sequence of GmWRKY21 was inserted into the sites of *Eco*RI and *Bam*HI of pGBKT7 vector to form the fusion carrier of GmWRKY21-pGBKT7. The fusion plasmid of GmWRKY21-pGBKT7 and pGBKT7 alone (negative control) were transformed into the cells of yeast strain Y2H. The cell deposits were colored with a chromogenic substrate of X-gal. The full coding sequence of GmWRKY21 (without TGA) was inserted into the *Nco*I and *Spe*I sites of pCAMBIA1302 vector to obtain the GmWRKY21-GFP fusion construct. The pCAMBIA1302-GFP vector was transformed as a control. Scale bar **(A,B)**: 10 μm.

To determine the transcriptional activity of GmWRKY21 protein, the complete GmWRKY21 ORF was inserted into the pGBKT7 vector. The results showed that the cell deposits transformed by the GmWRKY21-pGBKT7 construct turned blue on the SD/-Trp/X-α-Gal medium. However, the yeast cells transformed by the pGBKT7 vector did not turn blue (Figure 2A). These results indicated that the GmWRKY21 protein had transcriptional activity in yeast cells.

To investigate the subcellular localization of GmWRKY21 protein, the GmWRKY21-GFP (green fluorescent protein) recombinant was transformed into the cells of tobacco leaves. The results showed that the green fluorescence was observed in both cytoplasm and nucleus in the cells transformed with the pCAMBIA1302 vector, while the green fluorescence was only observed in the nucleus in the cells transformed with the GmWRKY21-pCAMBIA1302 vector (Figure 2B). The results indicated that the GmWRKY21 protein is localized in the nucleus.

### Expression Patterns of *GmWRKY21*

**Figure 3:**
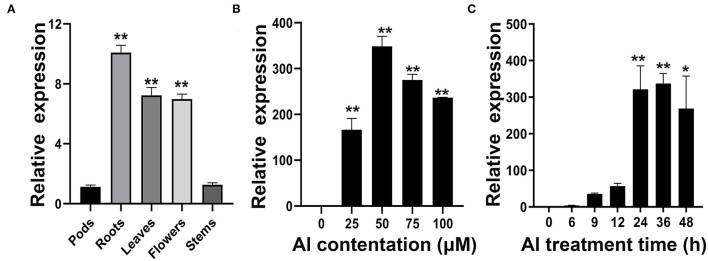
Analysis of *GmWRKY21* expression patterns in different tissues and in response to Al stress. **(A)** qRT-PCR analysis of the *GmWRKY21* transcript in different tissues of the soybean variety Huaxia 3. The total RNA was isolated from the samples of soybean roots, stems, leaves, flowers, and pods. **(B)** Dose-dependent *GmWRKY21* expression pattern in the roots of soybean seedlings. The root samples were taken from the seedlings exposed to different treatments of AlCl_3_ concentrations (0, 25, 50, 75, and 100 μM) for 24 h. **(C)** The time-course *GmWRKY21* expression pattern in the roots of soybean seedlings. The soybean seedlings were exposed to 50 μM AlCl_3_ for 0, 6, 9, 12, 24, 36, or 48 h. The root samples were separately harvested for qRT-PCR analysis. The expressing values are designed as the means ± SEM (*n* = 3). The experiments were performed with at least three independent biological replicates. Significant differences according to the one-way analysis of variance are denoted as follows: *P < 0.05 and **P < 0.01.

The qRT-PCR was used to investigate the expression patterns of *GmWRKY21*. The results showed that *GmWRKY21* presented a constitutive expression pattern, which was rich in soybean roots with about a 10-fold expression value, and more transcripts in leaves and flowers than those in stems or pods (Figure 3A). The *GmWRKY21* gene was upregulated under the dose treatments of AlCl_3_ with more than 160-fold relative expression values, and the *GmWRKY21* transcripts reached the maximum value under the treatment of 50 μM AlCl_3_ with about 350-fold relative expression value compared with that under the control treatment (Figure 3B). While under the time-course treatments of 50 μM AlCl_3_, the *GmWRKY21* gene was promptly upregulated with over 50-fold relative expression value and reached its much higher value after the treatment of 50 μM AlCl_3_ for 24 h (Figure 3C). After 50 μM AlCl_3_ treatment for 48 h, the expression of *GmWRKY21* was still at a relatively high level with over 250-fold relative expression (Figure 3C).

### *GmWRKY21* Transgenic Plants Confer Al Tolerance

**Figure 4:**
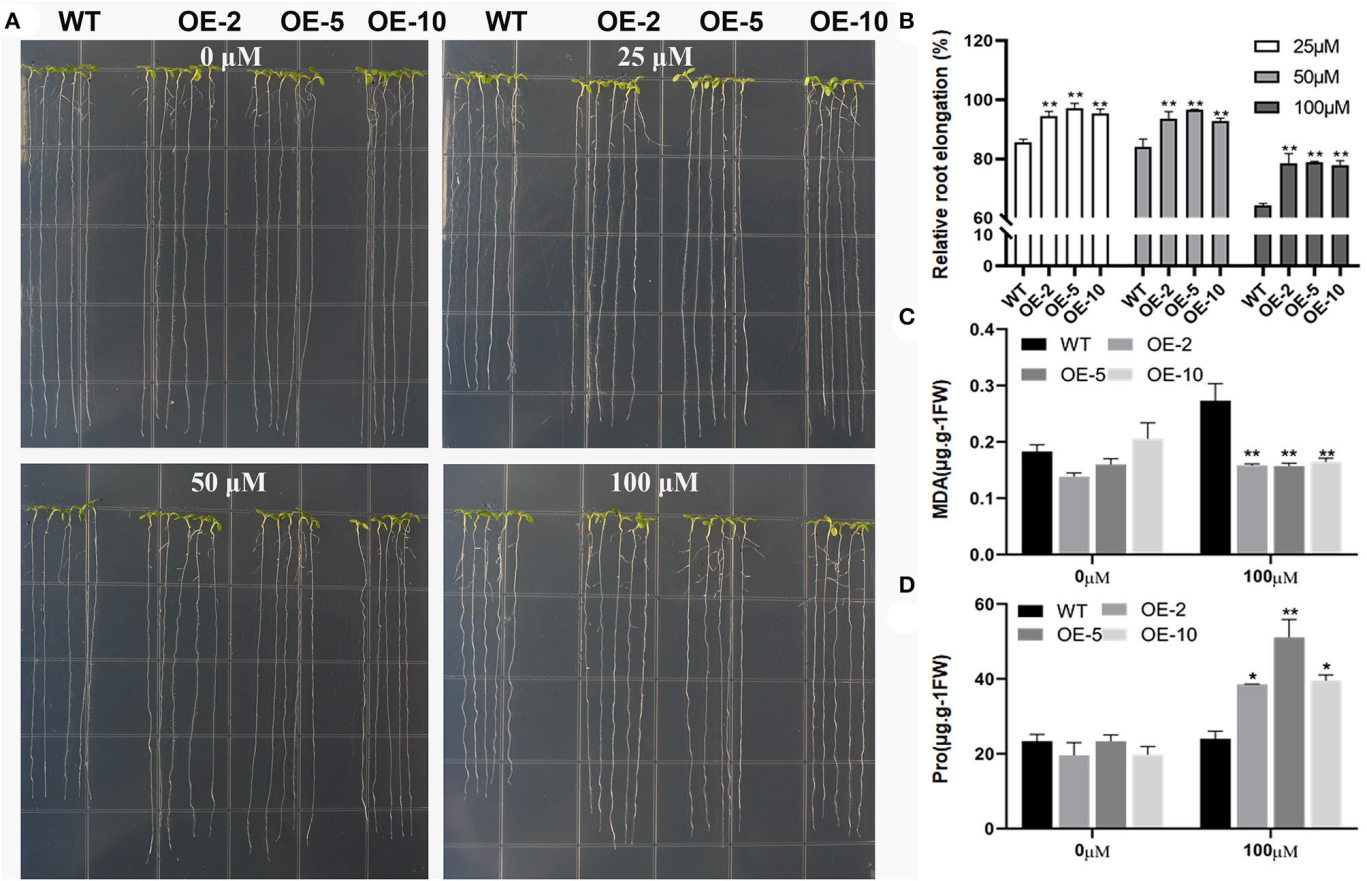
Overexpression of *GmWRKY21* conferred enhanced Al tolerance in transgenic Arabidopsis. **(A)** The phenotypes of *GmWRKY21* transgenic lines (OE-2, 5, and 10) and wild type (WT) under different treatments of AlCl_3_ solutions. Five-day-old seedlings were transferred to solid agar medium supplied with 1 mM CaCl_2_ and 1% sucrose containing different concentrations of AlCl_3_ (0, 25, 50, or 100 μM; pH 4.5) for 5 days. **(B)** The analysis of relative root elongation for the *GmWRKY21* transgenic lines and WT. **(C)** The determination of MDA content. **(D)** The determination of free proline content. All data are presented as means ± SEM. A significant difference according to the one-way analysis of variance is denoted as follows: *P < 0.05, **P < 0.01.

To investigate *GmWRKY21* response to Al stress, three homozygous lines (OE-2, OE-5, and OE-10) and wild type were used to observe the phenotypes under the treatments of AlCl_3_ in *Arabidopsis*. As shown in Figure 4A, no statistical difference was observed in root growth between WT and *GmWRKY21* transgenic lines in the absence of AlCl_3_. However, in the presence of 25 and 50 μM AlCl_3_, the root elongation of WT was inhibited by 15 and 16% after 5 days, but the root elongation of *GmWRKY21* transgenic lines was not significantly influenced. The root elongation of both WT and transgenic lines was inhibited with the increase in Al concentrations. At 100 μM AlCl_3_, the root elongation of WT was inhibited by 38% and that of the transgenic lines was inhibited by 12% (Figure 4B). The determination results showed that MDA was accumulated in both WT and *GmWRKY21* transgenic lines with 0.2733 and 0.1643 μg/g fresh weight, respectively (Figure 4C). In contrast, proline contents in both wild-type plants and *GmWRKY21* transgenic lines were 24 and 38–51 μg/g fresh weight, respectively, under the treatment of 100 μM AlCl_3_ (Figure 4D).

**Figure 5:**
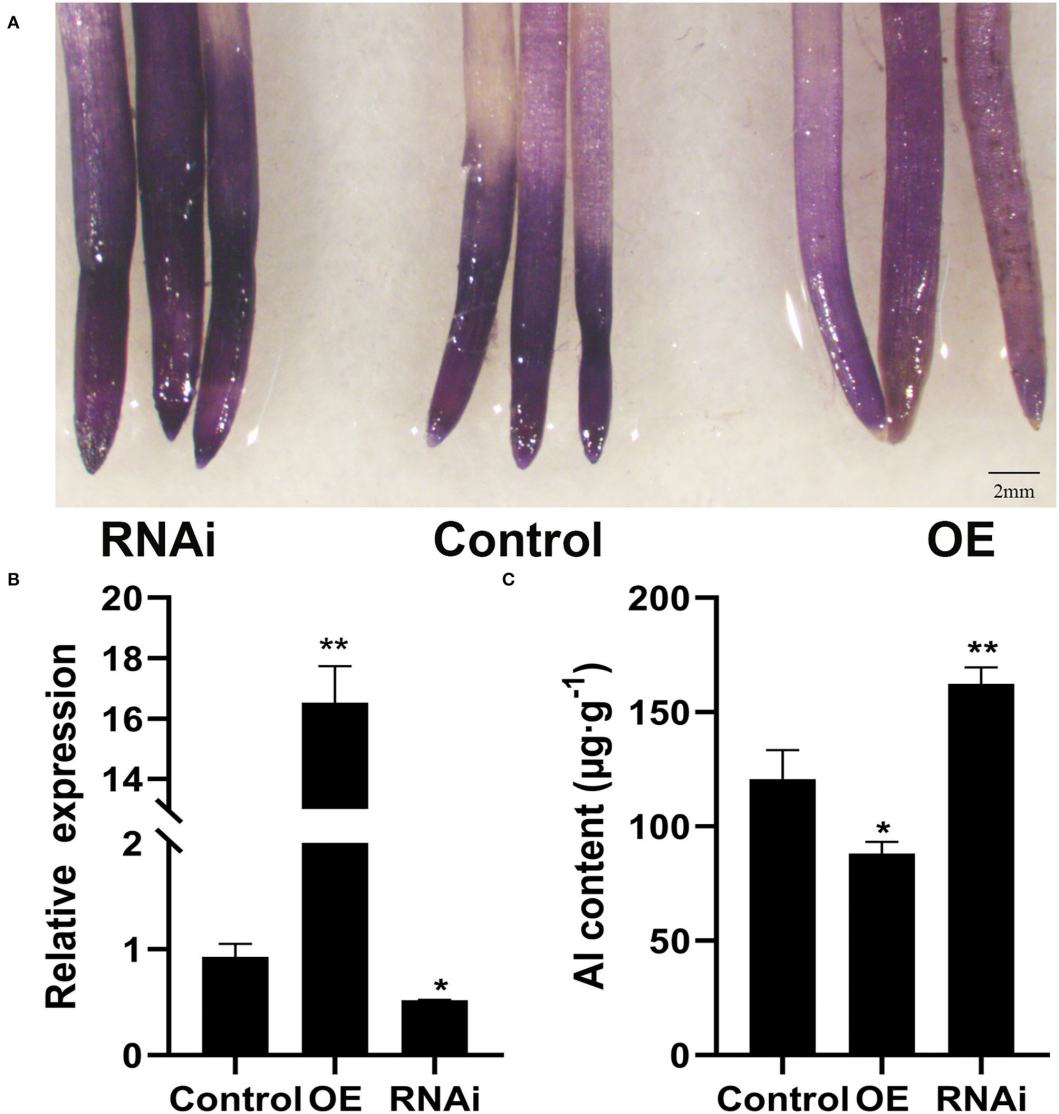
Hematoxylin staining in soybean hairy roots. **(A)** Hematoxylin staining in soybean hairy roots. **(B)** Detection of RNA level in soybean hairy roots. **(C)** Determination of Al^3+^ content in root tips. OE: the hairy roots of *GmWRKY21*-overexpressing transgenic soybean; RNAi: the hairy roots of *GmWRKY21*-RNAi transgenic soybean; Control: the hairy roots of Agrobacterium rhizogenes pathogenic strain K599 in soybean. The experiments were performed with at least three independent biological replicates. Significant differences according to the one-way analysis of variance are denoted as follows: *P < 0.05 and **P < 0.01.

To assess the effect of *GmWRKY21* overexpression on Al tolerance in soybean, transgenic hairy roots were produced using the A. rhizogenes-mediated transformation system (Figure 5A). Transcript analysis revealed that *GmWRKY21* was upregulated in the overexpression lines at an average fold of 16 and the expression of *GmWRKY21* was downregulated in the RNAi transgenic lines at an average fold of 0.5 (Figure 5B). Hematoxylin staining analysis of root tips showed that the *GmWRKY21*-silenced transgenic lines accumulated more Al^3+^ than those of the overexpression lines. The determination in root tips showed that the Al^3+^ contents were 88.1, 162.4, and 120.7 μg/g in the tips of OE, RNAi, and WT plants, respectively (Figure 5C). The results indicated that overexpression of *GmWRKY21* improves the tolerance of the hairy roots to Al stress by reducing Al^3+^ accumulation in soybean roots.

### Expression Patterns of Genes Responsive to Al Stress

**Figure 6:**
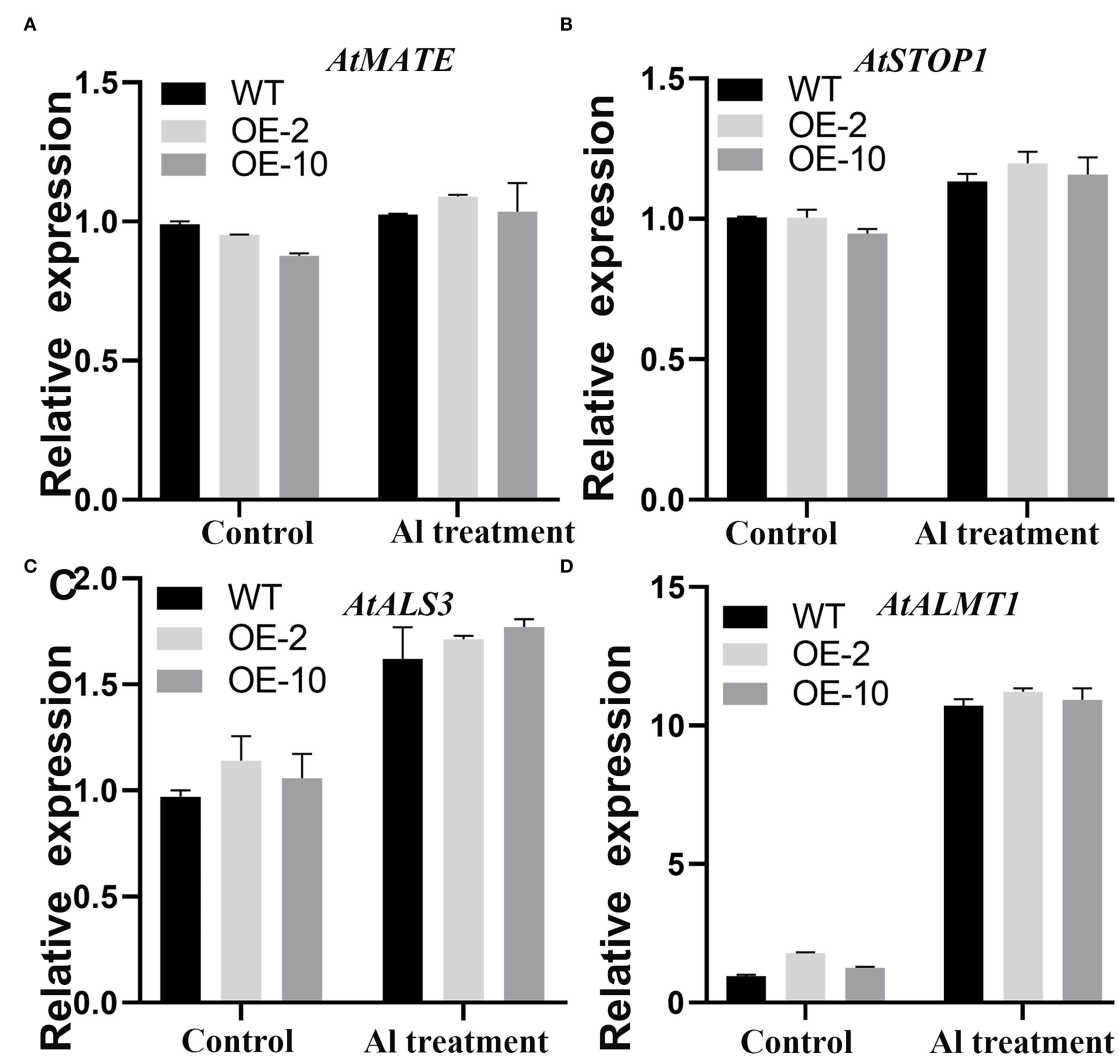
**(A–D)** Expression patterns of genes responsive to Al stress. WT: wild-type Arabidopsis; OE-2, OE-10: *GmWRKY21* overexpression transgenic lines. WT and transgenic *Arabidopsis* were grown on 0 or 100 μM AlCl_3_ (pH 4.3) 1/2 MS medium for about 10 days. All data are presented as means ± SEM (*n* = 3). Significant differences according to the one-way analysis of variance are denoted.

The qRT-PCR was carried out to analyze the molecular regulation mechanism of the *GmWRKY21* gene tolerant to acidic aluminum. The results showed that the expression of *AtALMT1, AtALS3, AtMATE*, and *AtSTOP1* was all upregulated under Al stress in transgenic Arabidopsis (Figure 6). The transcripts of *AtALMT1* and *AtALS3* induced by Al stress were much higher than those of them under the control; however, the expression of *AtMATE* and *AtSTOP1* was not significantly higher compared with that of the control (Figure 6). The results showed that *GmWRKY21* enhances the transgenic plants tolerant to Al stress mediated by the multiple genes through the pathway responding to aluminum toxicity.

### Expression Patterns of Stress-Responsive Genes Regulated by *GmWRKY21* in *Arabidopsis*

**Figure 7:**
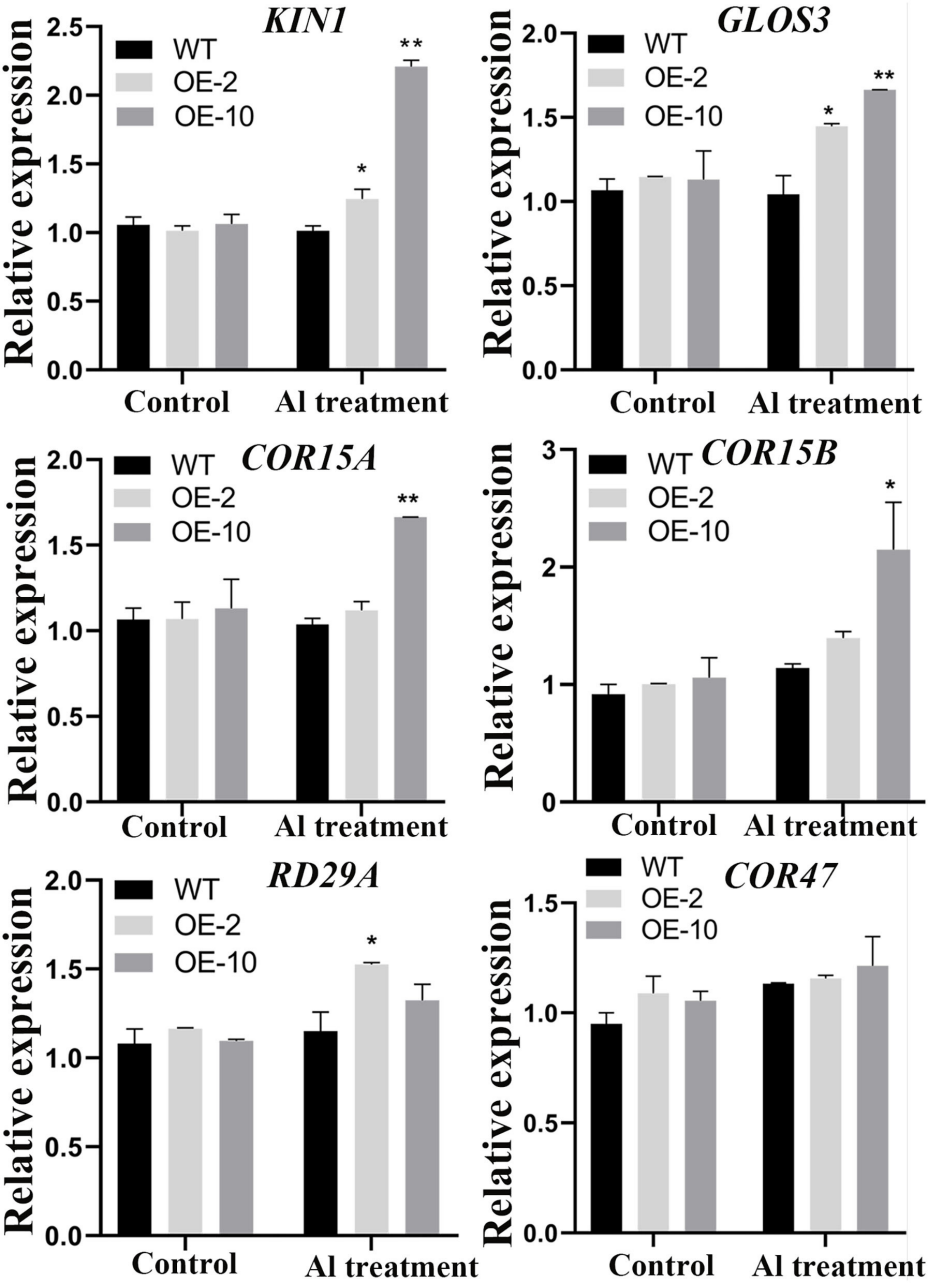
Expression patterns of stress-responsive genes regulated by *GmWRKY21*in Arabidopsis. WT, wild-type Arabidopsis; OE-2, OE-10: *GmWRKY21* overexpression transgenic lines. WT and transgenic *Arabidopsis* were grown on 0 or 100 μM AlCl_3_ (pH 4.3) 1/2 MS medium for about 10 days. All data are presented as means ± SEM (*n* = 3). Significant differences according to the one-way analysis of variance are denoted as follows: **P < 0.01 and *P < 0.05.

To further investigate the pathways regulated by *GmWRKY21* under Al stress, the expression patterns of stress-responsive genes were performed by qRT-PCR. The results showed that the stress-responsive genes, such as *KIN1, COR15A, COR15B, COR47, GLOS3*, and *RD29A*, were upregulated by the treatment of 100 μM AlCl_3_ (Figure 7). Among them, the transcripts of *KIN1, GLOS3, COR15A*, and *COR15B* genes were significantly higher in the OE-10 line than those of WT under Al stress. However, only the expression levels of *KIN1, GLOS3*, and *RD29A* were significantly different from those in the OE-2 lines. The results showed that the GmWRKY21 TF enhances the tolerance of transgenic *Arabidopsis* to acidic aluminum stress mediated by regulating the expression of the stress-responsive genes.

### *GmWRKY21* Transgenic Hairy Roots Increased Transcripts of Abiotic Stress-Related Marker Genes

**Figure 8:**
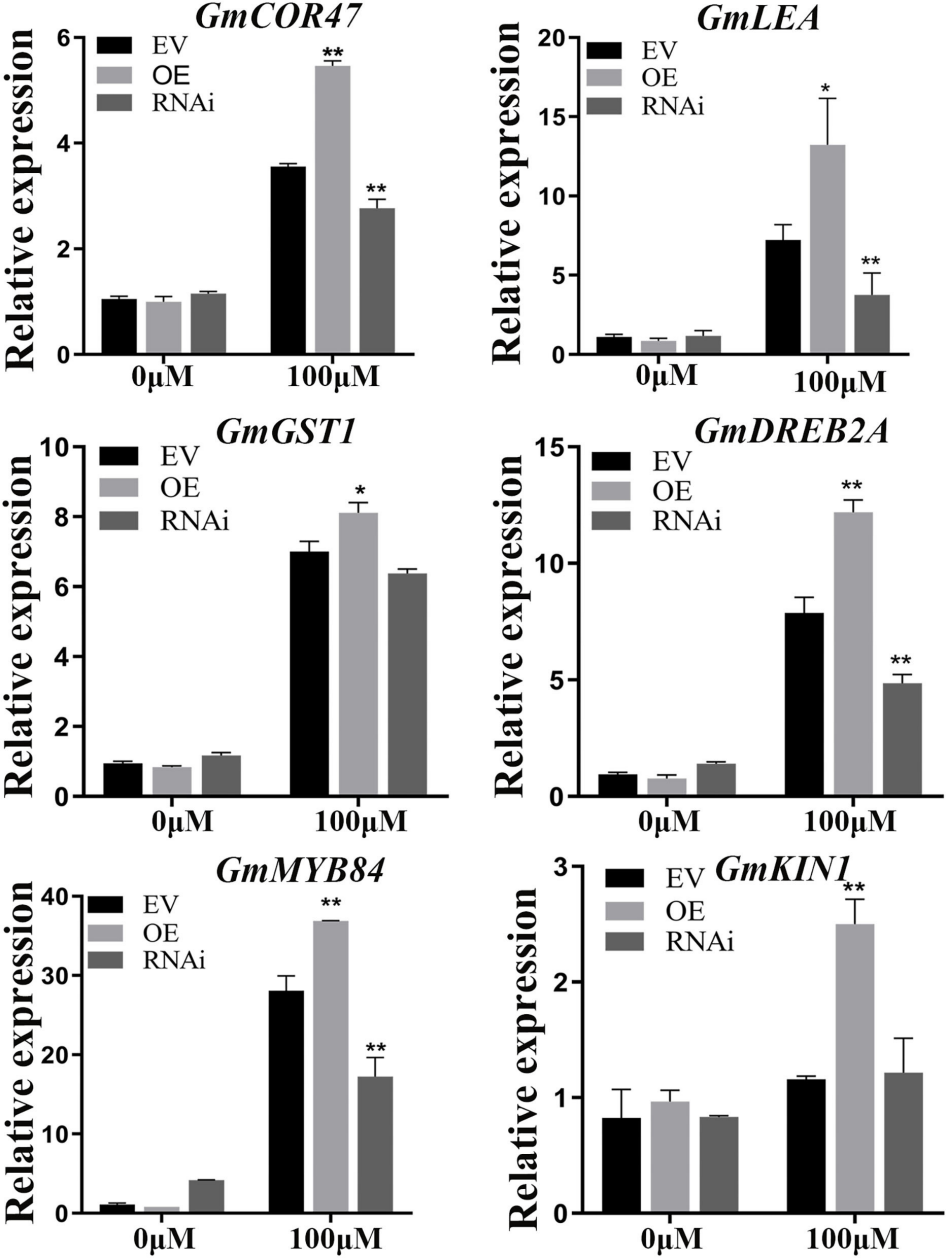
Expression patterns of the stress-responsive genes including *GmCOR47, GmLEA, GmGST1, GmDREB2A, GmMYB84*, and *GmKIN1*. EV, empty vector; OE, overexpressing *GmWRKY21* soybean hair roots; RNAi: the hairy roots of *GmWRKY21*-RNAi transgenic soybean. The EV, OE, and RNAi soybean hair roots under normal and Al stress conditions. The hair roots were exposed to 0 and 100 μM AlCl_3_ (pH 4.3) for 24 h. All data are presented as means ± SEM (*n* = 3). Significant differences according to the one-way analysis of variance are denoted as follows: **P < 0.01 and *P < 0.05.

Several genes such as *GmCOR47* (Guo et al., 1992), *GmDREB2A* (Agarwal et al., 2007), *GmMYB84* (Wang Y. Q. et al., 2017)*, GmKIN1* (Wang et al., 1995), *GmGST1* (Qi et al., 2018), and *GmLEA* (Magwanga et al., 2018) play significant roles in abiotic stress. The expression levels of these marker genes were analyzed between EV and *GmWRKY21* transgenic (OE or RNAi) hairy roots under normal and Al stress conditions by qRT-PCR analysis. There was no significant difference between the EV roots and either of the *GmWRKY21* transgenic soybean hairy roots under normal growth conditions. The expression levels of *GmCOR47, GmDREB2A, GmMYB84, GmKIN1, GmGST1*, and *GmLEA* in the OE soybean hairy roots were significantly higher than those in the EV roots. Conversely, the expression levels of these stress-responsive genes in the RNAi soybean hairy roots were significantly lower than those in the EV-control soybean hairy roots (Figure 8).

## Discussion

Since the first WRKY transcription factor gene *SPF1* was found in sweet potato in 1994 (Ishiguro and Nakamura, 1994), the WRKY family genes have been broadly investigated for the diverse biological roles in plant growth and development and responses to biotic and abiotic stress (Dai et al., 2016; Kiranmai et al., 2018). However, few genes are known about the WRKYs' roles and their mechanisms responsive to Al stress in soybean. In this study, the candidate gene *GmWRKY21* derived from the RIL (recombinant inbred line, F_12_) population of Zhonghuang 24 and Huaxia 3 (160 lines) was investigated for its functional characterization of tolerance to aluminum stress in Arabidopsis (Wang et al., 2019). The GmWRKY21 protein located in subgroup IIc contained the WRKY domain and C_2_H_2_-type zinc-finger structure with the classical characteristic of WRKY proteins from soybean and Arabidopsis (Figures 1A,B). The GmWRKY21 protein had more similarities to other WRKY proteins, such as GmWRKY6, GmWRKY25, GmWRKY45, GmWRKY53, GmWRKY8, GmWRKY54, and GmWRKY45, in soybean and AtWRKY57, AtWRKY45, AtWRKY71, AtWRKY28, AtWRKY75, and AtWRKY12 in Arabidopsis (Supplementary Figure 1). Previous reports indicated that these genes were all related to biotic and abiotic stress. For example, *GmWRKY6, GmWRKY25, GmWRKY53*, and *GmWRKY8* all respond to salt and drought stress (Zhou et al., 2008). *GmWRKY45* enhanced the adaptation to phosphate deficiency and salt tolerance in transgenic Arabidopsis (Zhou et al., 2008; Li et al., 2019; Wei et al., 2019). Overexpression of *GmWRKY54* resulted in the tolerance to salt and drought stress in Arabidopsis and the resistance to drought stress through activating genes in Ca^2+^ signaling pathways and abscisic acid in transgenic soybean (Wei et al., 2019). In addition, the WRKY family genes also play multiple roles in regulating plant growth and development, responding to biotic and abiotic stresses. Previous reports indicated that *AtWRKY57* conferred drought tolerance in Arabidopsis (Jiang et al., 2012). AtWRKY45 transcription factor activated *PHT1; 1* expression in response to phosphate starvation (Wang et al., 2014). *AtWRKY71* accelerated flowering in Arabidopsis in the presence of salt stress (Yu Z. Y. et al., 2018). Co-expression of *AtWRKY28* and *AtbHLH17* conferred resistance to abiotic stress in Arabidopsis (Babitha et al., 2013). AtWRKY75 interacted with DELLA protein to positively regulate Arabidopsis flowering (Zhang et al., 2018), while *AtWRKY12* negatively regulated cadmium tolerance in Arabidopsis by directly targeting GSH1 (Han et al., 2019). Furthermore, the phylogenetic tree indicated that the GmWRKY21 protein responding to salt and drought stress had more similarities to the GmWRKY6 protein (Supplementary Figure 1). Previous reports showed that overexpression of *GmWRKY21* responding to various abiotic stresses, such as salt and drought, enhanced the tolerance of low-temperature stress in transgenic Arabidopsis (Zhou et al., 2008). Therefore, the GmWRKY21 protein was identified to be a WRKY transcription factor to participate in abiotic stress and/or development in plants.

Previous studies revealed that WRKY TFs were widely investigated for key roles in acidic aluminum stress (Ding et al., 2013; Li et al., 2018, 2020). In this study, the *GmWRKY21* gene was rich in soybean root and quickly upregulated under AlCl_3_ treatment with the differences in Al^3+^ concentrations and temporal differences (Figure 3). Aluminum stress increased the formation of reactive oxygen species (ROS) in plants, leading to lipid membrane peroxidation, ion leakage, and protein oxidation (Ezaki et al., 2000; Boscolo et al., 2003). Malondialdehyde (MDA) was one of the most commonly used indicators for the destruction of ROS under stress conditions (Shi et al., 2013), while proline was a multifunctional amino acid to protect plant growth by accumulating in large quantities when plants were subjected to adversity stress (Natarajan et al., 2012). In this study, overexpression of *GmWRKY21* enhanced the tolerance of transgenic Arabidopsis and soybean hairy roots to acid–aluminum stress with the corresponding changes of MDA and proline (Figures 4, 6). Hematoxylin was easy to combine with the root aluminum to form a purplish red complex, and the root tip aluminum ion content can be indirectly judged by the depth of root tip color so as to obtain the sensitivity of plants to aluminum toxicity (Rincon and Gonzales, 1992). In this study, the lines of *GmWRKY21* overexpression were dyed shallower by hematoxylin staining with less Al^3+^ content in the root tips compared to those of WT and RNAi plants (Figure 5). Recent reports indicated that other WRKY TFs also have the function of coping with acidic aluminum stress. For example, *AtWRKY46* was a transcriptional suppressor of *ALMT1* that regulates alumina-induced Arabidopsis malic acid secretion. The mutation of *AtWRKY46* led to increased malic acid secretion and reduced aluminum accumulation in the root tip resulting in higher aluminum tolerance in Arabidopsis (Ding et al., 2013). *OsWRKY22* enhanced the tolerance to aluminum stress by activating the expression of *OsFRDL4* and enhancing the secretion of citric acid in rice (Li et al., 2018). *AtWRKY47* made Arabidopsis aluminum tolerant by regulating cell wall modification genes (Li et al., 2020). Therefore, the *GmWRKY21* gene may play an important role in the response of plants to acidic aluminum stress.

Previous studies have found that the ALMT protein family can transport malic acid in the root system to the rhizosphere conferring plants tolerant to aluminum toxicity (Sasaki et al., 2004). The MATE protein family was also involved in the aluminum rejection mechanism by transporting citric acid from the root system to the rhizosphere (Ryan et al., 2011; Kochian et al., 2015). In Arabidopsis, *ALMT1, MATE*, and *ALS3* were upregulated by the STOP1 transcription factor (Sawaki et al., 2009). In this study, the *STOP1, ALMT1, ALS3*, and *MATE* genes were upregulated under aluminum acid stress, while the *STOP1, ALMT1*, and *ALS3* genes were dramatically induced by *GmWRKY21* in transgenic lines. However, the transcripts of the four genes in transgenic Arabidopsis under Al stress were statistically non-significant differences compared to those under the control (Figure 6). The results suggested that *GmWRKY21* enhances the tolerance to Al stress in transgenic plants by the comprehensive effect of multiple genes. Previous research found that *GmWRKY21* was involved in a variety of abiotic stresses, such as low-temperature, salt, and drought stress (Zhou et al., 2008). In this study, the stress-related genes of *KIN1, GLOS3*, and *RD29A* were significantly upregulated in transgenic Arabidopsis plants under the AlCl_3_ treatment (Figure 7; Yu et al., 2016; Chu et al., 2018; Wang et al., 2018; Ma et al., 2019). The LEA genes of *COR15A* and *COR15B* were upregulated with more transcripts in transgenic Arabidopsis plants (Figure 7), which were abundantly present in vegetative plant tissues under drought, low-temperature or salt, and other environmental stresses (Hundertmark and Hincha, 2008). Overexpression of *COR15A* or *COR15B* significantly improved the freezing resistance of Arabidopsis, while silencing these two genes at the same time reduced the freezing resistance of Arabidopsis (Artus et al., 1996; Thalhammer et al., 2014). The results indicated that *GmWRKY21* enhanced the tolerance to aluminum stress in Arabidopsis by several genes mediated by certain stress-responsive pathways.

A previous study indicated that some genes such as *GmCOR47, GmMYB84, GmDREB2A, GmGST1, GmKIN1*, and *GmLEA* play certain roles in regulating plant response to abiotic stress by potential mechanism. *GmGST1* helps in maintaining redox homeostasis of cells, thus enhancing plant ability to resist abiotic stress (Qi et al., 2018). *GmMYB84* can improve the expression levels of *GmRBOHB-1* and *GmRBOHB-2* genes by binding to the MBS cis-elements in their promoter, enhancing stress tolerance in soybean (Wang N. et al., 2017). The COR47 and LEA as group 2 LEA (LEA II) proteins encode dehydrins, which are cold-inducible proteins that are supposed to sustain membrane stabilization and prevent protein aggregation (Guo et al., 1992; Yu Y. C. et al., 2018; Yu Z. Y. et al., 2018; Li P. et al., 2021). The DREB protein plays an important role in regulating abiotic stress-related genes and thereby imparting stress tolerance to the plant system (Dubouzet et al., 2003). *KIN1* was induced by low temperature, ABA, osmoticum, and dehydration (Wang et al., 1995). These stress-responsive genes were significantly upregulated in the hairy root of transgenic *GmWRKY21* soybean (Figure 8). This indicates that *GmWRKY21* improved soybean resistance to aluminum stress by regulating the expression of several stress-responsive genes.

## Data Availability Statement

The original contributions presented in the study are included in the article/Supplementary Material, further inquiries can be directed to the corresponding author/s.

## Author Contributions

QM, HN, and ZH conceived and designed the study. ZH, JW, XW, XZ, YC, and ZC conducted the experiments. ZH, JW, and QM performed the data and statistical analysis. ZH wrote the manuscript which was reviewed and edited by HN and QM. All authors contributed to the article and approved the submitted version.

## Funding

This work was supported by grants from the National Natural Science Foundation of China (31971965 and 31771816), the Major Project of New Varieties Cultivation of Genetically Modified Organisms (2016ZX08004002-007), the Special Supervision on Quality and Safety of Agricultural Products of the Ministry of Agriculture and Rural Areas (4100-C17106 and 21301091702101), the Key Projects of International Scientific and Technological Innovation Cooperation among Governments under National Key R&D Plan (2018YFE0116900-06), the Key Area Research and Development Program of Guangdong Province (2020B020220008), the China Agricultural Research System (CARS-04-PS09), and the Project of Science and Technology of Guangzhou (201804020015).

## Conflict of Interest

The authors declare that the research was conducted in the absence of any commercial or financial relationships that could be construed as a potential conflict of interest.

## Publisher's Note

All claims expressed in this article are solely those of the authors and do not necessarily represent those of their affiliated organizations, or those of the publisher, the editors and the reviewers. Any product that may be evaluated in this article, or claim that may be made by its manufacturer, is not guaranteed or endorsed by the publisher.
