# Supplementary material for: GmWRKY21, a Soybean WRKY Transcription Factor Gene, Enhances the Tolerance to Aluminum Stress in Arabidopsis thaliana
Source: Front Plant Sci. 2022 Jul 25;13:833326. doi: 10.3389/fpls.2022.833326 (PMC9359102; doi:10.3389/fpls.2022.833326)
Supplement: Supplementary file 1 [file Data_Sheet_1.docx]

Supplementary Material

**1 Supplementary Figure and Table**

- 1. **Supplementary Figure**


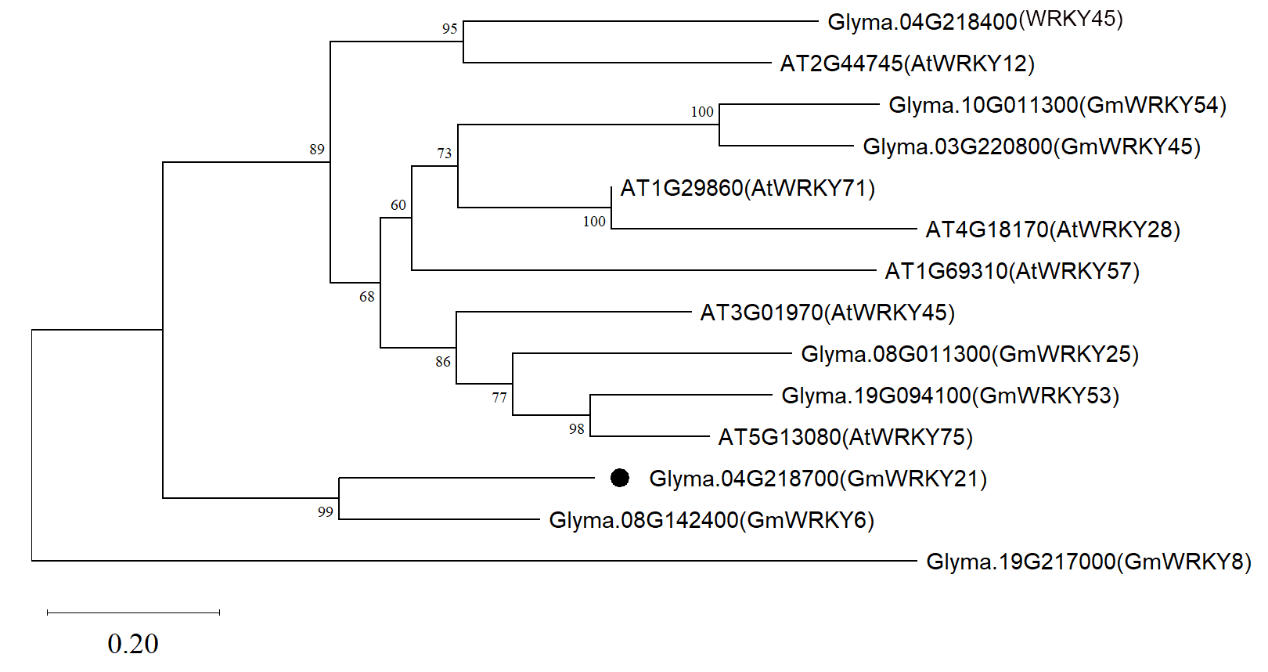


**Supplementary Figure S1.** Phylogenetic tree analysis of GmWRKY21 and the published genes in group Ⅱ-c of WRKY transcription factor family. The GmWRKY21 gene was labeled with a black circle. The detailed information of the published genes in group Ⅱ-c of WRKY transcription factor family were available from the Supplementary Table S2.


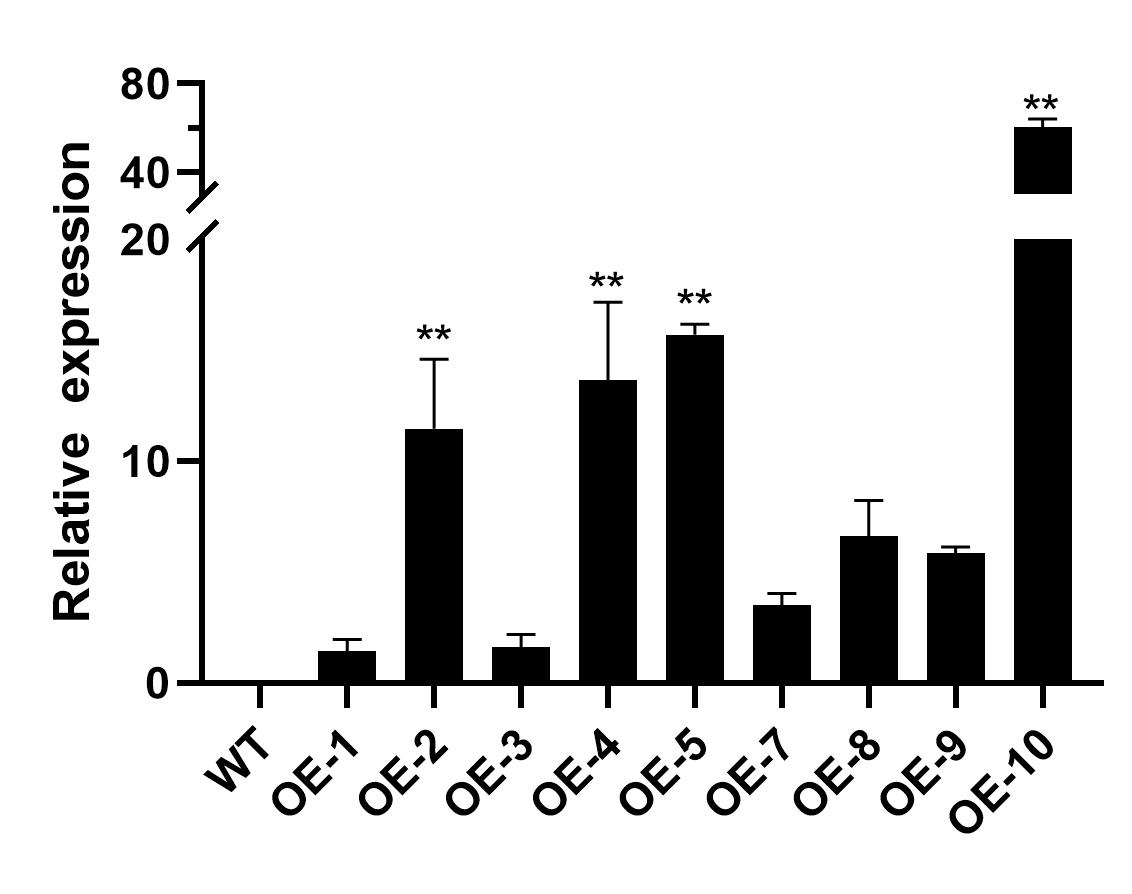


**Supplementary Figure S2.** Molecular identification of *GmWRKY21* transgenic Arabidopsis lines. qRT-PCR identification of *GmWRKY21* transgenic Arabidopsis lines. The three-week-old seedlings were used to perform the molecular identification of *GmWRKY21* transgenic Arabidopsis plant and lines. WT: wild type; OE-1, 2, 3, 4, 5, 7, 8, 9, and 10: *GmWRKY21* transgenic Arabidopsis lines of T_3_ generation. *GmWRKY21* transcription levels were substantially (*P* < 0.01) higher in OE-2, OE-4, OE-5 and OE-10 than in WT.

­

**1.2 Supplementary Table 1**

| **Primer names** | **Primer sequences (5'to3')** |
| --- | --- |
| GmWRKY21-F | CTTGGGTCTTTGGCTCTT |
| GmWRKY21-R | GGATCATGAGTTTGCAGAAG |
| pTF101-WRKY-F | gagaacacgggggactctagaATGGATTACTATTTTGGAAACCCTAAT |
| pTF101-WRKY-R | cgatcggggaaattcgagctcTCATGAGTTTGCAGAAGGGTGC |
| pGBKT7W21-F | atggccatggaggccgaattcATGGATTACTATTTTGGAAACCCTAAT |
| pGBKT7W21-R | ccgctgcaggtcgacggatccCTGAGTTTGCAGAAGGGTGCA |
| p1302-WRKY-F | acgggggactcttgaccatggCTATGGATTACTATTTTGGAAACCCTAAT |
| p1302-WRKY-R | aagttcttctcctttactagtTGAGTTTGCAGAAGGGTGCAG |
| Actin3-F | GCACCACCGGAGAGAAAATA |
| Actin3-R | GTGCACAATTGATGGACCAG |
| MATE-qRT-F | GCATAGGACTTCCGTTTGTGGCA |
| MATE-qRT-R | CGAACACAAACGCTAAGGCA |
| RD29A-F | GGCGTAACAGGTAAACCTAGAG |
| RD29A-R | TCCGATGTAAACGTCGTCC |
| ALMT-qRT-F | ACTTGAGAGAGCTGAGTGACC |
| ALMT-qRT-R | TCTTCTCGGGTCTTCATTCCC |
| AtSTOP1-qF1 | TTTCCGCGACTGATGTTTGAT |
| AtSTOP1-qR1 | ACAGGCATTCGCAATAAGCAT |
| COR15A-F | GGCCACAAAGAAAGCTTCAG |
| COR15A-R | CTTGTTTGCGGCTTCTTTTC |
| KIN1-F | AACAAGAATGCCTTCCAAGC |
| KIN1-R | CGCATCCGATACACTCTTTCC |
| GLOS3-F | GGAGTGGTTGGTCTGGCTAA |
| GLOS3-R | TTGGTTATCCGGTGGGTAAA |
| COR15B-F | TCAGTGGCATGGGTTCTTCTTCCA |
| COR15B-R | GAGGTCATCGAGGATGTTGCCGT |
| COR47-F | CAGTGTCGGAGAGTGTGGTG |
| COR47-R | ACAGCTGGTGAATCCTCTGC |
| pGBKT7-F | ATCATGGAGGAGCAGAAG |
| pGBKT7-R | GGGGTTATGCTAGTTATG |
| p1302-F | TGTGAAGATAGTGGAAAAG |
| p1302-R | GCCACGGAACAGGTAGTTT |

**2 Supplementary Data**

**2.1 CDS sequencing information of *G*m*WRKY21***

ATGGATTACTATTTTGGAAACCCTAATCCTAAACCTTATGATAACCGTCACTCTGCCGTAGTGAACACGGAATCTCCTTCCTCCGAGTTCATGCTATCTGATTATCTCGTGTTGGAAGATGCTGTCGATAATCAAGAGTCTTGGTCACAAAGCACTGAAACTGAATCATCGGAGAAAGGAAACTCCAGCGATGTCAGTCATGGGTTTGGTGATGCAACCTTCAGCAACACCAACATGCATATAAAGTGCGAAAATAATGGGATAAAGCGAAAGAAGGAAGAAGTGAGTCAAATGATCACGTTTAGAACCAGATCGCAGCTTGAGGTTATGGATGATGGATATAAATGGAGGAAATACGGAAAGAAGACAGTGAAGAACAATCCCAACCCAAGGAACTACTACAAGTGTTCAGGTGAAGGATGCAATGTGAAGAAAAGGGTGGAAAGAGACAGGGATGACTCGAACTATGTTTTAACAACGTACGACGGTGTCCACAATCATGAGAGCCCGTCGACTGCCTACTACAGCCAAATTCCCTTGGTGCATTCTAACCATGATTGGCCCCAGCTGCACCCTTCTGCAAACTCATGA

**2.2 Amino acid sequence of GmWRKY21 protein**

MDYYFGNPNPKPYDNRHSAVVNTESPSSEFMLSDYLVLEDAVDNQESWSQSTETESSEKGNSSDVSHGFGDATFSNTNMHIKCENNGIKRKKEEVSQMITFRTRSQLEVMDDGYKWRKYGKKTVKNNPNPRNYYKCSGEGCNVKKRVERDRDDSNYVLTTYDGVHNHESPSTAYYSQIPLVHSNHDWPQLHPSANS*
