# Supplementary material for: GmWRKY21, a Soybean WRKY Transcription Factor Gene, Enhances the Tolerance to Aluminum Stress in Arabidopsis thaliana
Source: Front Plant Sci. 2022 Jul 25;13:833326. doi: 10.3389/fpls.2022.833326 (PMC9359102; doi:10.3389/fpls.2022.833326)
Supplement: Supplementary file 2 [file Table_1.docx]

**Table 2. The information of *WRKY* genes from Arabidopsis and soybean**

| **Serial number** | **Gene** | **Gene ID** | **Descriptions** | **References** |
| --- | --- | --- | --- | --- |
| 1 | *Glyma.01G053800* | 100127369 |  |  |
| 2 | *Glyma.01G043300* | 100797911 |  |  |
| 3 | *Glyma.01G056800* | 100127427 |  |  |
| 4 | *Glyma.01G128100* | 100127367 |  |  |
| 5 | *Glyma.01G189100* | 100804089 |  |  |
| 6 | *Glyma.01G222300* | 102667019 |  |  |
| 7 | *Glyma.01G224800* | 100127370 | Improve Drought and Salt Tolerance | Shi, W., Du, Y., Ma, J, et al. The WRKY transcription factor GmWRKY12 confers drought and salt tolerance in soybean [J]. International Journal of Molecular Sciences, 2018 19(12), 4087. |
| 8 | *Glyma.02G007500* | 100785933 |  |  |
| 9 | *Glyma.02G010900* | 100784158 |  |  |
| 10 | *Glyma.02G020300* | 100809801 |  |  |
| 11 | *Glyma.02G112100* | 100799335 |  |  |
| 12 | *Glyma.02G115200* | 100170676 |  |  |
| 13 | *Glyma.02G141000* | 100170681 |  |  |
| 14 | *Glyma.02G203800* | 100782194 |  |  |
| 15 | *Glyma.02G232600* | 100127385 |  |  |
| 16 | *Glyma.02G285900* | 100790175 |  |  |
| 17 | *Glyma.02G293400* | 102667867 |  |  |
| 18 | *Glyma.02G297400* | 100816583 |  |  |
| 19 | *Glyma.02G306300* | 100782726 |  |  |
| 20 | *Glyma.03G042700* | 102666898 |  |  |
| 21 | *Glyma.03G109100* | 100788263 |  |  |
| 22 | *Glyma.03G159700* | 100127372 |  |  |
| 23 | *Glyma.03G176600* | 100127379 |  |  |
| 24 | *Glyma.03G220100* | 100127423 |  |  |
| 25 | *Glyma.03G220800* | 732590 | Response to salt and drought stresses | Zhou Q Y, Tian A G, Zou H F, et al. Soybean WRKY‐type transcription factor genes, *GmWRKY13*, *GmWRKY21*, and GmWRKY54, confer differential tolerance to abiotic stresses in transgenic Arabidopsis plants [J]. Plant biotechnology journal, 2008, 6(5): 486-503. |
| 26 | *Glyma.03G224700* | 100817839 |  |  |
| 27 | *Glyma.03G256700* | 100127387 |  |  |
| 28 | *Glyma.04G054200* | 100819462 |  |  |
| 29 | *Glyma.04G061300* |  |  |  |
| 30 | *Glyma.04G061400* | 100798500 |  |  |
| 31 | *Glyma.04G076200* | 100127394 |  |  |
| 32 | *Glyma.04G115500* | 100796545 |  |  |
| 33 | *Glyma.04G173500* |  |  |  |
| 34 | *Glyma.04G218400* | 100127389 | Response to salt stress | Zhou Q Y, Tian A G, Zou H F, et al. Soybean WRKY‐type transcription factor genes, *GmWRKY13*, *GmWRKY21*, and *GmWRKY54*, confer differential tolerance to abiotic stresses in transgenic Arabidopsis plants [J]. Plant biotechnology journal, 2008, 6(5): 486-503. |
| 35 | *Glyma.04G218700* | 732584 | Improve Freezing Tolerance | Zhou Q Y, Tian A G, Zou H F, et al. Soybean WRKY‐type transcription factor genes, *GmWRKY13*, *GmWRKY21*, and *GmWRKY54*, confer differential tolerance to abiotic stresses in transgenic Arabidopsis plants [J]. Plant biotechnology journal, 2008, 6(5): 486-503. |
| 36 | *Glyma.04G223200* | 100792150 | Regulate flowering time | Yang Y, Chi Y, Wang Z, et al. Functional analysis of structurally related soybean GmWRKY58 and GmWRKY76 in plant growth and development [J]. Journal of experimental botany, 2016, 67(15): 4727-4742. |
| 37 | *Glyma.04G238300* | 100776906 |  |  |
| 38 | *Glyma.05G029000* | 100793946 |  |  |
| 39 | *Glyma.05G096500* | 100170685 |  |  |
| 40 | *Glyma.05G123000* | 100820209 |  |  |
| 41 | *Glyma.05G123600* | 100776919 |  |  |
| 42 | *Glyma.05G127600* | 100787050 |  |  |
| 43 | *Glyma.05G185400* |  |  |  |
| 44 | *Glyma.05G165800* |  |  |  |
| 45 | *Glyma.05G160800* | 100793777 |  |  |
| 46 | *Glyma.05G184500* | 100500248 |  |  |
| 47 | *Glyma.05G211900* | 102663255 |  |  |
| 48 | *Glyma.05G215900* | 100815760 |  |  |
| 49 | *Glyma.06G054500* | 100808823 |  |  |
| 50 | *Glyma.06G061900* | 100127373 |  |  |
| 51 | *Glyma.06G077400* | 100170746 |  |  |
| 52 | *Glyma.06G125600* | 100812027 |  |  |
| 53 | *Glyma.06G142000* | 100778162 | Regulate flowering time | Yang Y, Chi Y, Wang Z, et al. Functional analysis of structurally related soybean GmWRKY58 and GmWRKY76 in plant growth and development [J]. Journal of experimental botany, 2016, 67(15): 4727-4742. |
| 54 | *Glyma.06G142100* | 100786198 |  |  |
| 55 | *Glyma.06G147100* | 100127396 |  |  |
| 56 | *Glyma.06G147500* | 100799241 |  |  |
| 57 | *Glyma.06G168400* | 100796070 |  |  |
| 58 | *Glyma.06G190800* | 100804403 |  |  |
| 59 | *Glyma.06G212900* | 100817211 |  |  |
| 60 | *Glyma.06G219800* | 100800480 |  |  |
| 61 | *Glyma.06G307700* | 100795177 |  |  |
| 62 | *Glyma.06G320700* | 100127395 |  |  |
| 63 | *Glyma.07G023300* | 100807966 |  |  |
| 64 | *Glyma.07G057400* | 100127428 |  |  |
| 65 | *Glyma.07G116300* | 100800671 |  |  |
| 66 | *Glyma.07G133700* | 100810459 |  |  |
| 67 | *Glyma.07G227200* | 100802109 |  |  |
| 68 | *Glyma.07G238000* | 100788697 |  |  |
| 69 | *Glyma.07G262700* | 100127382 |  |  |
| 70 | *Glyma.08G011300* | 100127378 | Response to salt and drought stresses | Zhou Q Y, Tian A G, Zou H F, et al. Soybean WRKY‐type transcription factor genes, *GmWRKY13*, *GmWRKY21*, and *GmWRKY54*, confer differential tolerance to abiotic stresses in transgenic Arabidopsis plants [J]. Plant biotechnology journal, 2008, 6(5): 486-503. |
| 71 | *Glyma.08G018300* | 102667104 |  |  |
| 72 | *Glyma.08G021900* | 100127375 |  |  |
| 73 | *Glyma.08G078100* |  |  |  |
| 74 | *Glyma.08G078700* | 102669548 |  |  |
| 75 | *Glyma.08G082400* | 100816891 |  |  |
| 76 | *Glyma.08G118200* | 100127392 |  |  |
| 77 | *Glyma.08G142400* | 732583 | Response to salt and drought stresses | Zhou Q Y, Tian A G, Zou H F, et al. Soybean WRKY‐type transcription factor genes, *GmWRKY13*, *GmWRKY21*, and *GmWRKY54*, confer differential tolerance to abiotic stresses in transgenic Arabidopsis plants [J]. Plant biotechnology journal, 2008, 6(5): 486-503. |
| 78 | *Glyma.08G143400* | 732585 | Enhance the Resistance to *Phytophthora sojae* | Cui, X., Yan, Q., Gan, S. et al. *GmWRKY40*, a member of the WRKY transcription factor genes identified from *Glycine max* L., enhanced the resistance to *Phytophthora sojae*. BMC Plant Biol, 2019, 19, 598. |
| 79 | *Glyma.08G218600* | 100170678 |  |  |
| 80 | *Glyma.08G240800* | 100170684 |  |  |
| 81 | *Glyma.08G320200* | 100804246 |  |  |
| 82 | *Glyma.08G325800* | 100127383 |  |  |
| 83 | *Glyma.09G005700* | 100127376 |  |  |
| 84 | *Glyma.09G029800* | 100805707 |  |  |
| 85 | *Glyma.09G034300* | 100127422 |  |  |
| 86 | *Glyma.09G061900* | 100127391 |  |  |
| 87 | *Glyma.09G080000* | 100816719 |  |  |
| 88 | *Glyma.09G127100* |  |  |  |
| 89 | *Glyma.09G129100* | 100788213 |  |  |
| 90 | *Glyma.09G240000* | 100792084 |  |  |
| 91 | *Glyma.09G244000* | 100526878 |  |  |
| 92 | *Glyma.09G250500* | 100813723 |  |  |
| 93 | *Glyma.09G254400* | 100796481 |  |  |
| 94 | *Glyma.09G254800* | 100798084 |  |  |
| 95 | *Glyma.09G274000* | 100805895 |  |  |
| 96 | *Glyma.09G280200* | 100776837 |  |  |
| 97 | *Glyma.10G2* | 732591 | Improve Drought Tolerance | Wei W, Liang DW, Bian XH. et al. GmWRKY54 improves drought tolerance through activating genes in abscisic acid and Ca^2+^ signaling pathways in transgenic soybean. Plant J. 2019, 100(2):384-398. |
| 98 | *Glyma.10G032900* | 100792310 |  |  |
| 99 | *Glyma.10G113800* |  |  |  |
| 100 | *Glyma.10G138300* | 100780044 |  |  |
| 101 | *Glyma.10G171000* | 102666131 |  |  |
| 102 | *Glyma.10G171200* |  |  |  |
| 103 | *Glyma.10G171100* | 102666270 |  |  |
| 104 | *Glyma.10G230200* | 100127366 |  |  |
| 105 | *Glyma.11G021200* | 102669888 |  |  |
| 106 | *Glyma.11G053100* | 100127371 |  |  |
| 107 | *Glyma.11G163300* | 100127421 |  |  |
| 108 | *Glyma.12G097100* | 100797998 |  |  |
| 109 | *Glyma.12G152600* | 100127388 |  |  |
| 110 | *Glyma.12G212300* | 100170680 |  |  |
| 111 | *Glyma.13G102000* | 732587 | Less Sensitive to ABA | Zhou Q Y, Tian A G, Zou H F, et al. Soybean WRKY-type transcription factor genes, *GmWRKY13*, *GmWRKY21*, and *GmWRKY54*, confer differential tolerance to abiotic stresses in transgenic Arabidopsis plants [J]. Plant biotechnology journal, 2008, 6(5): 486-503. |
| 112 | *Glyma.13G117600* | 100797109 |  |  |
| 113 | *Glyma.13G267400* | 102663141 |  |  |
| 114 | *Glyma.13G267500* | 102663281 |  |  |
| 115 | *Glyma.13G267600* | 100792193 |  |  |
| 116 | *Glyma.13G267700* | 102659367 |  |  |
| 117 | *Glyma.13G289400* | 100127424 |  |  |
| 118 | *Glyma.13G310100* | 100127384 |  |  |
| 119 | *Glyma.13G370100* | 100798375 |  |  |
| 120 | *Glyma.14G006800* | 100781219 |  |  |
| 121 | *Glyma.14G016200* | 547723 |  |  |
| 122 | *Glyma.14G028900* | 100806016 |  |  |
| 123 | *Glyma.14G102900* | 100791870 |  |  |
| 124 | *Glyma.14G103100* | 100810978 |  |  |
| 125 | *Glyma.14G135400* | 100127381 |  |  |
| 126 | *Glyma.14G185800* | 100796071 |  |  |
| 127 | *Glyma.14G186000* | 100796599 |  |  |
| 128 | *Glyma.14G186100* | 100797129 |  |  |
| 129 | *Glyma.14G199800* | 102667674 |  |  |
| 130 | *Glyma.14G200200* | 100127393 |  |  |
| 131 | *Glyma.15G003300* | 732588 |  |  |
| 132 | *Glyma.15G110300* | 100802124 |  |  |
| 133 | *Glyma.15G135600* | 100804773 |  |  |
| 134 | *Glyma.15G139000* | 100812252 |  |  |
| 135 | *Glyma.15G168200* | 100127386 |  |  |
| 136 | *Glyma.15G186300* | 100781603 |  |  |
| 137 | *Glyma.16G026400* | 100170747 |  |  |
| 138 | *Glyma.16G031400* | 100810669 |  |  |
| 139 | *Glyma.16G031900* | 100815829 |  |  |
| 140 | *Glyma.16G054400* | 100788382 |  |  |
| 141 | *Glyma.16G176700* | 100794708 |  |  |
| 142 | *Glyma.16G177000* | 100796829 |  |  |
| 143 | *Glyma.16G219800* | 102670495 |  |  |
| 144 | *Glyma.17G011400* | 100784668 |  |  |
| 145 | *Glyma.17G035400* | 100808190 |  |  |
| 146 | *Glyma.17G042300* | 100779314 |  |  |
| 147 | *Glyma.17G057100* | 732589 |  |  |
| 148 | *Glyma.17G074000* | 100127377 |  |  |
| 149 | *Glyma.17G097900* | 100805360 |  |  |
| 150 | *Glyma.17G168900* | 100788748 |  |  |
| 151 | *Glyma.17G197500* |  |  |  |
| 152 | *Glyma.17G222300* | 100127380 |  |  |
| 153 | *Glyma.17G222500* | 100170679 |  |  |
| 154 | *Glyma.17G224800* | 100796836 |  |  |
| 155 | *Glyma.17G239200* | 100786245 |  |  |
| 156 | *Glyma.18G056600* | 100127397 |  |  |
| 157 | *Glyma.18G081200* | 100781491 |  |  |
| 158 | *Glyma.18G092200* | 100779533 |  |  |
| 159 | *Glyma.18G124700* | 102666229 |  |  |
| 160 | *Glyma.18G183100* | 100803946 |  |  |
| 161 | *Glyma.18G208800* | 100792833 |  |  |
| 162 | *Glyma.18G213200* | 100170682 |  |  |
| 163 | *Glyma.18G238200* | 100170675 |  |  |
| 164 | *Glyma.18G238600* | 100127398 |  |  |
| 165 | *Glyma.18G242000* | 100800211 |  |  |
| 166 | *Glyma.18G256500* | 100780402 |  |  |
| 167 | *Glyma.18G263400* | 100796679 |  |  |
| 168 | *Glyma.19G020600* | 100819296 |  |  |
| 169 | *Glyma.19G094100* | 732586 | Response to salt and drought stresses | Zhou Q Y, Tian A G, Zou H F, et al. Soybean WRKY‐type transcription factor genes, *GmWRKY13*, *GmWRKY21*, and *GmWRKY54*, confer differential tolerance to abiotic stresses in transgenic Arabidopsis plants [J]. Plant biotechnology journal, 2008, 6(5): 486-503. |
| 170 | *Glyma.19G177400* | 100794069 |  |  |
| 171 | *Glyma.19G217000* | 100127368 | Response to salt and drought stresses | Zhou Q Y, Tian A G, Zou H F, et al. Soybean WRKY‐type transcription factor genes, *GmWRKY13*, *GmWRKY21*, and *GmWRKY54*, confer differential tolerance to abiotic stresses in transgenic Arabidopsis plants [J]. Plant biotechnology journal, 2008, 6(5): 486-503. |
| 172 | *Glyma.19G217800* | 100816452 |  |  |
| 173 | *Glyma.19G221700* | 100783661 |  |  |
| 174 | *Glyma.19G254800* | 100790050 |  |  |
| 175 | *Glyma.20G028000* | 100784936 |  |  |
| 176 | *Glyma.20G030500* |  |  |  |
| 177 | *Glyma.20G163200* | 100792164 |  |  |
| 178 | *AT2G04880* | 815035 | Regulate Stomatal Movement in Drought stress | Qiao, Z., Li, CL. & Zhang, W. WRKY1 regulates stomatal movement in drought-stressed *Arabidopsis thaliana*. Plant Mol Biol, 2016, 91: 53–65. |
| 179 | *AT1G55600* | 842009 |  |  |
| 180 | *AT4G31550* | 829282 | Response to Abiotic Stress | Ali MA, Azeem F, Nawaz MA, et al. Transcription factors WRKY11 and WRKY17 are involved in abiotic stress responses in Arabidopsis [J]. J Plant Physiology, 2018, 226: 12-21. |
| 181 | *AT2G44745* | 819083 | Negatively Regulate Cadmium Tolerance | Han, Y., Fan, T., Zhu, X. et al. WRKY12 represses *GSH1* expression to negatively regulate cadmium tolerance in *Arabidopsis* [J]. Plant Mol Biol, 2019, 99: 149–159. |
| 182 | *AT4G39410* | 830096 | Positively Regulate Cadmium Tolerance | Ren Y, Fan T, Xiao F, et al. The WRKY transcription factor, WRKY13, activates *PDR8* expression to positively regulate cadmium tolerance in *Arabidopsis*. Plant Cell Environ [J]. 2019, 42(3):891-903. |
| 183 | *AT1G30650* | 839945 |  |  |
| 184 | *AT2G23320* | 816864 |  |  |
| 185 | *AT5G45050* | 834536 |  |  |
| 186 | *AT2G24570* | 816993 | Response to Abiotic Stress | Ali MA, Azeem F, Nawaz MA, et al. Transcription factors WRKY11 and WRKY17 are involved in abiotic stress responses in *Arabidopsis* [J]. J Plant Physiology, 2018, 226: 12-21. |
| 187 | *AT4G31800* | 829308 |  |  |
| 188 | *AT4G12020* | 826810 |  |  |
| 189 | *AT5G56270* | 835726 |  |  |
| 190 | *AT4G26640* | 828771 |  |  |
| 191 | *AT2G30590* | 817609 |  |  |
| 192 | *AT4G01250* | 827896 |  |  |
| 193 | *AT2G47260* | 819339 |  |  |
| 194 | *AT5G41570* | 834159 |  |  |
| 195 | *AT2G30250* | 817575 | Response to Heat Stress | Li, S., Fu, Q., Huang, W. et al. Functional analysis of an *Arabidopsis* transcription factor *WRKY25* in heat stress [J]. Plant Cell Rep, 2009), 28: 683–693. |
| 196 | *AT5G07100* | 830601 | Response to Heat Tolerant | Li S, Fu Q, Chen L, et al. *Arabidopsis thaliana* WRKY25, WRKY26, and WRKY33 coordinate induction of plant thermotolerance [J]. Planta, 2011, 233(6): 1237-1252. |
| 197 | *AT5G52830* | 835360 |  |  |
| 198 | *AT4G18170* | 827542 | Response to Drought Tolerant | Babitha K C, Ramu S V, Pruthvi V, et al. Co-expression of *AtbHLH17* and *AtWRKY28* confers resistance to abiotic stress in *Arabidopsis* [J]. Transgenic research, 2013, 22(2): 327-341. |
| 199 | *AT4G23550* | 828455 |  |  |
| 200 | *AT2G03340* | 814863 |  |  |
| 201 | *AT5G24110* | 832476 |  |  |
| 202 | *AT4G22070* | 828296 |  |  |
| 203 | *AT4G30935* | 829218 |  |  |
| 204 | *AT2G38470* | 818429 |  |  |
| 205 | *AT4G26440* | 828750 |  |  |
| 206 | *AT2G34830* | 818048 |  |  |
| 207 | *AT1G69810* | 843317 |  |  |
| 208 | *AT5G22570* | 832320 |  |  |
| 209 | *AT3G04670* | 819625 |  |  |
| 210 | *AT1G13960* | 837956 |  |  |
| 211 | *AT1G80840* | 844423 |  |  |
| 212 | *AT4G11070* | 826708 |  |  |
| 213 | *AT4G04450* | 825775 |  |  |
| 214 | *AT2G46130* | 819220 |  |  |
| 215 | *AT2G37260* | 818303 |  |  |
| 216 | *AT3G01970* | 821270 | Response to Phosphate Starvation | Wang H, Xu Q, Kong Y H, et al. Arabidopsis WRKY45 transcription factor activates *PHOSPHATE TRANSPORTER1;1* expression in response to phosphate starvation [J]. Plant Physiology, 2014, 164(4):2020-2029. |
| 217 | *AT2G46400* | 819248 |  |  |
| 218 | *AT4G01720* | 828001 | Involve in Al Tolerance | Li CX, Yan JY, Ren JY, et al. A WRKY transcription factor confers aluminum tolerance via regulation of cell wall modifying genes [J]. J Integr Plant Biol, 2020, 62(8):1176-1192. |
| 219 | *AT5G49520* | 835012 |  |  |
| 220 | *AT5G43290* | 834347 |  |  |
| 221 | *AT5G26170* | 832686 |  |  |
| 222 | *AT5G64810* | 836602 |  |  |
| 223 | *AT5G45260* | 834562 |  |  |
| 224 | *AT4G23810* | 828481 | Negatively Regulate Drought Tolerance | Sun Y, Yu D. Activated expression of *AtWRKY53* negatively regulates drought tolerance by mediating stomatal movement [J]. Plant Cell Rep, 2015, 34(8):1295-306. |
| 225 | *AT2G40750* | 818670 | Modulate OsmoticStress Tolerance | Li J, Besseau S, Törönen P, et al. Defense-related transcription factors WRKY70 and WRKY54 modulate osmotic stress tolerance by regulating stomatal aperture in *Arabidopsis* [J]. New Phytol, 2013, 200(2):457-472. |
| 226 | *AT1G64000* | 842703 |  |  |
| 227 | *AT1G69310* | 843262 | Improve Drought Tolerance | Jiang Y, Liang G, Yu D. Activated expression of WRKY57 confers drought tolerance in Arabidopsis [J]. Mol Plant, 2012, 5(6):1375-88. |
| 228 | *AT3G01080* | 821213 |  |  |
| 229 | *AT2G21900* | 816726 |  |  |
| 230 | *AT1G62300* | 842527 |  |  |
| 231 | *AT2G25000* | 817039 |  |  |
| 232 | *AT1G18860* | 838467 |  |  |
| 233 | *AT5G01900* | 831826 |  |  |
| 234 | *AT1G66600* | 842978 | Response to Abscisic Acid and Drought Tolerance | Ren X, Chen Z, Liu Y, Zhang H, et al. ABO3, a WRKY transcription factor, mediates plant responses to abscisic acid and drought tolerance in *Arabidopsis* [J]. Plant J, 2010, 63(3):417-29. |
| 235 | *AT1G66560* | 842974 |  |  |

| 236 | *AT1G29280* | 839802 |  |  |
| --- | --- | --- | --- | --- |
| 237 | *AT1G80590* | 844398 |  |  |
| 238 | *AT1G66550* | 842973 |  |  |
| 239 | *AT3G62340* | 825407 |  |  |
| 240 | *AT3G58710* | 825040 |  |  |
| 241 | *AT4G24240* | 828525 |  |  |
| 242 | *AT3G56400* | 824807 |  |  |
| 243 | *AT1G29860* | 839864 | Response to salt tolerant | Yu Y, Wang L, Chen J, et al. WRKY71 acts antagonistically against salt-delayed flowering in Arabidopsis thaliana [J]. Plant and Cell Physiology, 2018, 59(2): 414-422. |
| 244 | *AT5G15130* | 831365 |  |  |
| 245 | *AT5G28650* | 832971 |  |  |
| 246 | *AT5G13080* | 831147 | Regulate Flowering | Zhang L, Chen L, Yu D. Transcription factor WRKY75 interacts with DELLA proteins to affect flowering [J]. Plant Physiol, 2018, 176(1):790-803. |
| 247 | *AT5G46350* | 834678 | Modulate Salinity Stress Tolerance | Hu Y, Chen L, Wang H, et al. Arabidopsis transcription factor WRKY8 functions antagonistically with its interacting partner VQ9 to modulate salinity stress tolerance [J]. Plant J, 2013, 74(5):730-45. |
| 248 | *AT1G68150* | 843143 |  |  |
